# Artificial intelligence-enhanced electrocardiography to predict regurgitant valvular heart diseases: an international study

**DOI:** 10.1093/eurheartj/ehaf448

**Published:** 2025-07-16

**Authors:** Yixiu Liang, Arunashis Sau, Boroumand Zeidaabadi, Joseph Barker, Konstantinos Patlatzoglou, Libor Pastika, Ewa Sieliwonczyk, Zachary Whinnett, Nicholas S Peters, Ziqing Yu, Xi Liu, Shuo Wang, Hongyang Lu, Daniel B Kramer, Jonathan W Waks, Yangang Su, Junbo Ge, Fu Siong Ng

**Affiliations:** Department of Cardiology, Zhongshan Hospital of Fudan University, Shanghai, Institute of Cardiovascular Diseases, National Clinical Research Centre for Interventional Medicine, Shanghai 200032, China; National Heart and Lung Institute, Imperial College London, Hammersmith Hospital, Du Cane Road, London W12 0NN, UK; National Heart and Lung Institute, Imperial College London, Hammersmith Hospital, Du Cane Road, London W12 0NN, UK; Department of Cardiology, Imperial College Healthcare NHS Trust, London W12 0NN, UK; National Heart and Lung Institute, Imperial College London, Hammersmith Hospital, Du Cane Road, London W12 0NN, UK; National Heart and Lung Institute, Imperial College London, Hammersmith Hospital, Du Cane Road, London W12 0NN, UK; Department of Cardiology, Imperial College Healthcare NHS Trust, London W12 0NN, UK; National Heart and Lung Institute, Imperial College London, Hammersmith Hospital, Du Cane Road, London W12 0NN, UK; National Heart and Lung Institute, Imperial College London, Hammersmith Hospital, Du Cane Road, London W12 0NN, UK; National Heart and Lung Institute, Imperial College London, Hammersmith Hospital, Du Cane Road, London W12 0NN, UK; MRC Laboratory of Medical Sciences, Imperial College London, London, UK; University of Antwerp and Antwerp University Hospital, Antwerp, Belgium; National Heart and Lung Institute, Imperial College London, Hammersmith Hospital, Du Cane Road, London W12 0NN, UK; Department of Cardiology, Imperial College Healthcare NHS Trust, London W12 0NN, UK; National Heart and Lung Institute, Imperial College London, Hammersmith Hospital, Du Cane Road, London W12 0NN, UK; Department of Cardiology, Imperial College Healthcare NHS Trust, London W12 0NN, UK; Department of Cardiology, Zhongshan Hospital of Fudan University, Shanghai, Institute of Cardiovascular Diseases, National Clinical Research Centre for Interventional Medicine, Shanghai 200032, China; Department of Cardiology, Zhongshan Hospital of Fudan University, Shanghai, Institute of Cardiovascular Diseases, National Clinical Research Centre for Interventional Medicine, Shanghai 200032, China; School of Basic Medical Sciences, Digital Medical Research Center, Fudan University, Shanghai, China; Imperial College London, Data Science Institute, London SW7 2AZ, UK; Cardiac Rhythm Management, Medtronic Technology Center, Medtronic (Shanghai) Ltd, Shanghai, China; Richard A. and Susan F. Smith Center for Outcomes Research in Cardiology, Beth Israel Deaconess Medical Center, Harvard Medical School, Boston, MA, USA; Harvard Medical School, Beth Israel Deaconess Medical Center, Harvard-Thorndike Electrophysiology Institute, Boston, MA, USA; Department of Cardiology, Zhongshan Hospital of Fudan University, Shanghai, Institute of Cardiovascular Diseases, National Clinical Research Centre for Interventional Medicine, Shanghai 200032, China; Department of Cardiology, Zhongshan Hospital of Fudan University, Shanghai, Institute of Cardiovascular Diseases, National Clinical Research Centre for Interventional Medicine, Shanghai 200032, China; National Heart and Lung Institute, Imperial College London, Hammersmith Hospital, Du Cane Road, London W12 0NN, UK; Department of Cardiology, Imperial College Healthcare NHS Trust, London W12 0NN, UK; Department of Cardiology, Chelsea and Westminster NHS Foundation Trust, London SW10 9NH, UK

**Keywords:** Artificial intelligence, Electrocardiogram, Valvular heart disease

## Abstract

**Background and Aims:**

Valvular heart disease (VHD) is a significant source of morbidity and mortality, though early intervention can improve outcomes. This study aims to develop artificial intelligence-enhanced electrocardiography (AI-ECG) models to diagnose and predict future moderate or severe regurgitant VHDs (rVHDs), including mitral regurgitation (MR), tricuspid regurgitation (TR), and aortic regurgitation (AR).

**Methods:**

The AI-ECG models were developed in a data set of 988 618 ECG and transthoracic echocardiogram pairs from 400 882 patients from Zhongshan Hospital, Shanghai, China. The AI-ECG models used a residual convolutional neural network with a discrete-time survival loss function. External evaluation was performed in outpatients from a secondary care data set from Beth Israel Deaconess Medical Center, Boston, USA, consisting of 34 214 patients with linked echocardiography.

**Results:**

In the internal test set, the AI-ECG models accurately predicted future significant MR [*C*-index 0.774, 95% confidence interval (CI) 0.753–0.792], AR (0.691, 95% CI 0.657–0.720), and TR (0.793, 95% CI 0.777–0.808). In age- and sex-adjusted Cox models, the highest risk quartile had a hazard ratio (HR) of 7.6 (95% CI 5.8–9.9, *P* < .0001) for risk of future significant MR, compared with the lowest risk quartile. For future AR and TR, the equivalent HRs were 3.8 (95% CI 2.7–5.5) and 9.9 (95% CI 7.5–13.0), respectively. These findings were confirmed in the transnational external test set. Imaging association analyses demonstrated AI-ECG predictions were associated with subclinical chamber remodelling.

**Conclusions:**

This study developed AI-ECG models to diagnose and predict rVHDs and validated the models in a transnational and ethnically distinct cohort. The AI-ECG models could be utilized to guide surveillance echocardiography in patients at risk of future rVHDs, to facilitate early detection and intervention.

## Structured Graphical Abstract

**Structured Graphical Abstract:**
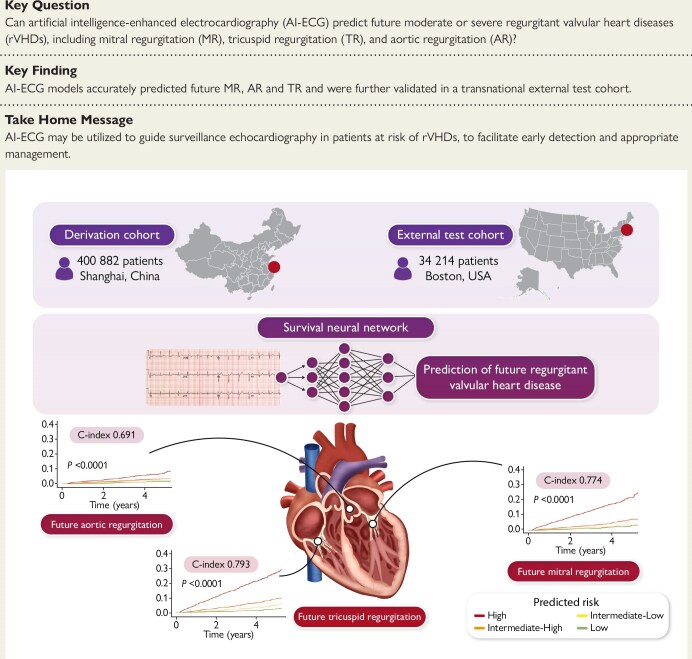
Artificial intelligence-enhanced electrocardiography predicts future regurgitant valvular heart disease in cohorts from the USA and China. USA: United States of America.

**See the editorial comment for this article ‘The electrocardiogram reimagined: AI-ECG as a prognostic beacon for valvular heart disease’, by A.A. Armoundas, https://doi.org/10.1093/eurheartj/ehaf584.**

## Introduction

Valvular heart disease (VHD) affects 41 million people worldwide, with increasing prevalence and associated mortality rates.^1^ Timely medical treatment as well as surgical or transcatheter intervention for clinically significant, i.e. moderate or severe VHD, has been shown to reduce mortality and improve outcomes.^2,3^ Consequently, it is important to identify patients who, although not currently exhibiting clinically significant VHD, are at high risk of developing it in the future.

Predicting the progression of significant VHD, however, remains a complex challenge. Demographic and echocardiographic predictors of VHD progression have been investigated,^4–6^ though these studies employed varying definitions of progression and no systematic predictive models have been developed. As a result, current clinical guidelines recommend regular echocardiographic surveillance unselectively for patients with mild VHDs,^7^ which can place a strain on limited healthcare resources, while no recommendations are provided for individuals without evidence of VHD.

Recently, artificial intelligence (AI)-enhanced electrocardiography (AI-ECG) models have demonstrated promise in the screening and diagnosis of prevalent significant VHDs,^8–11^ though no models currently exist for prediction of future significant VHD. Interestingly, false-positive AI-ECG predictions for prevalent VHD are independently associated with future risk of VHD progression during follow-up,^8,10^ suggesting that subtle ECG signatures of VHD may predate structural changes and therefore be important prognostic markers. Furthermore, wearable devices have recently been developed to enable continuous monitoring of ECG signals, facilitating early disease detection and prediction, which could enhance the management of VHD.^12^

We hypothesized that an AI-ECG model can not only accurately diagnose VHD but also predict future progression to significant VHD. Such models could help to tailor the frequency of echocardiographic surveillance based on an individualized risk of VHD progression. We trained and tested a survival-based deep learning model to predict the progression of the most prevalent regurgitant VHDs (rVHDs):^13^ mitral regurgitation (MR), aortic regurgitation (AR), and tricuspid regurgitation (TR), with external evaluation in a transnational and ethnically diverse cohort.

## Methods

### Data source

Data from two independent cohorts from China and the USA were utilized. The Shanghai Zhongshan Hospital (SHZS) cohort, which served as the derivation cohort, comprises all patients aged 14 years or older (the age at which assessment is conducted in the adult hospital) who underwent at least one 12-lead ECG and transthoracic echocardiography (TTE) at Zhongshan Hospital, Shanghai, China, between January 2013 and May 2020. The Beth Israel Deaconess Medical Center (BIDMC) cohort from Boston, USA, used as the external test cohort, consists of routinely collected data from patients over 16 years old with a valid ECG performed between 2000 and 2023, including ECGs recorded during outpatient encounters. These cohorts include a diverse range of individuals, from healthy patients to patients with various medical conditions. This spectrum encompasses both asymptomatic individuals undergoing routine assessments (in SHZS) and those with cardiac or non-cardiac conditions, reflecting a population more representative of the general public. Supplementary data online, *Tables S1* and *S2* and Supplementary Methods provide further details of the SHZS population.

The 12-lead ECG data were pre-processed using a bandpass filter ranging from 0.5 to 100 Hz and a notch filter at 60 Hz, followed by resampling to 400 Hz. We aimed for our model to reflect real-world clinical practice, acknowledging that ECG signal quality may not always be optimal. We therefore did not exclude ECGs due to poor quality or artefact. Zero padding was added to adjust the input shape to a power of 2, resulting in 4096 samples per lead for a 10-s recording, which served as the input to the neural network model. For TTEs, data on valvular regurgitation were extracted from the final echocardiographer diagnosis reports in tabular format. In both centres, echocardiographers followed the American Society of Echocardiography recommendations for the evaluation of valvular regurgitation^14^ and used an integrated approach including qualitative, semiquantitative, and quantitative metrics to assess the severity of the valvular regurgitation. Unfortunately, we were unable to assess what proportion of cases had quantitative metrics used to assess valve lesion severity. The diagnosis of rVHDs was then recalibrated to a scale of none, mild, moderate, and severe by recording the least severe assessment in the diagnostic statement. This approach is consistent with the approach of previous AI-ECG VHD studies.^10,11^ Patients with a history of cardiac valve surgery, a prosthetic valve, or pacemaker/defibrillator implantation were excluded for model evaluation, but included in model training as these patients may provide helpful additional training data even though there may be some noise introduced by including these conditions. A sensitivity analysis was performed to formally demonstrate that exclusion of these conditions resulted in similar or inferior performance (see Supplementary data online, *Table S3*). Electrocardiography and TTE data were first paired if the interval between the two examinations was no more than 60 days. Pairs with the shortest time interval between ECG and TTE were then preserved to ensure only one TTE was paired to each ECG.

**Figure 1:**
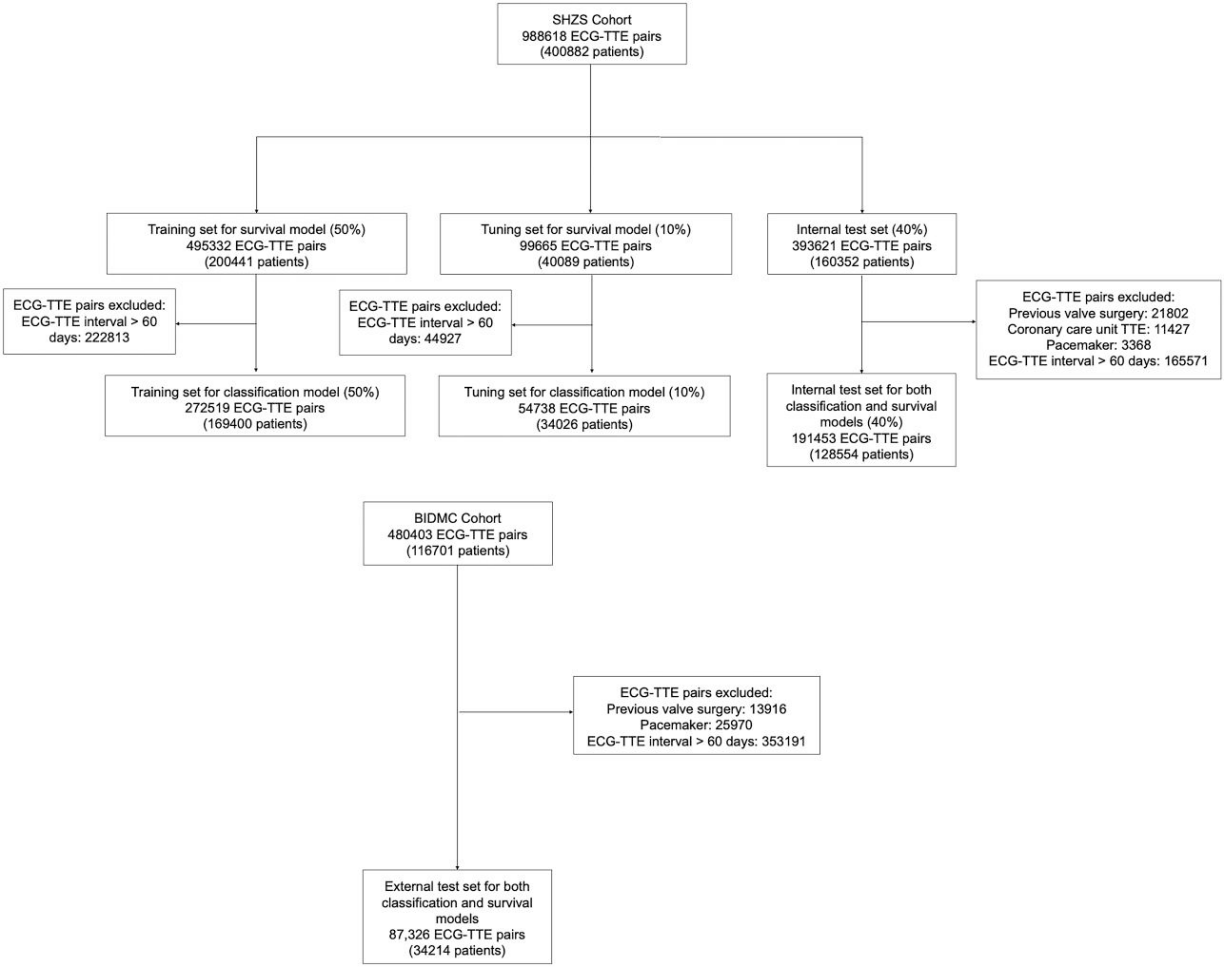
Study flow diagram. Diagram depicts data used to train and evaluate the artificial intelligence-enhanced electrocardiography survival and classification models. ECG, electrocardiogram; TTE, transthoracic echo; SHZS, Shanghai Zhongshan Hospital; BIDMC, Beth Israel Deaconess Medical Center

The data in the SHZS cohort were randomly split at a ratio of 50%, 10%, and 40% for training, tuning, and internal test sets, respectively (*Figure 1*). None of the patients were assigned to more than one group. The data in the BIDMC cohort were utilized as the external test set. Multiple ECGs per patient were used for model training in the SHZS cohort, while the first ECG per patient was used for evaluation of model performance in the internal test set of the SHZS cohort and external test set of the BIDMC cohort. Multiple ECGs per patient were used in the test sets to model the change in predictions over time and for explainability analyses.

All study procedures were performed in compliance with the Declaration of Helsinki. The study is reported in line with the Transparent Reporting of a multivariable prediction model for Individual Prognosis Or Diagnosis + AI (TRIPOD-AI) guidelines.^15^

### Model development

The outcome of the present study was the progression to clinically significant rVHD, defined as an increase in recorded TR, MR, or AR grade to moderate or severe during follow-up.^10,11,16,17^ Separate models were developed for MR, AR, and TR. Models were developed using two approaches, employing a previously described convolutional neural network (CNN) architecture based on residual blocks as the backbone.^18^ First, classification models were developed using a binary label of significant regurgitant MR, AR, or TR using ECGs with linked TTE within 60 days at baseline (training set *n* = 272 519 ECG–TTE pairs from 169 400 patients). Second, survival models were developed to both diagnose and predict future rVHD simultaneously, this approach allows the model to be trained using ECG-TTE pairs regardless of the time interval between the ECG and the TTE, as the time interval between ECG and TTE is accounted for during model training, and therefore, all available ECG–TTE pairs in the training/tuning sets were used (training set *n* = 495 332 ECG–TTE pairs from 200 441 patients, *Figure 1*). We adapted the final layer of the CNN backbone to accommodate a discrete-time survival approach.^19^ The discrete-time survival approach allows the model to account for both time to outcome and censorship. This approach was modified to encode both prevalent and future disease by encoding prevalent moderate or severe MR, AR, or TR at the first timepoint in the discrete-time survival labels. When evaluating predictions, outputs from the first timepoint were used to evaluate diagnostic tasks, while the 5-year timepoint was used for predictive tasks (details in Supplementary data online, Supplementary Methods).

### Model evaluation

Diagnostic performance was evaluated using the area under the receiver operating characteristic curve (AUROC), sensitivity, specificity, positive predictive value, negative predictive value, and area under the precision–recall curve. Calibration was assessed using the Brier score. The performance of the models for prediction of future rVHD was assessed using the Harrell’s concordance index (*C*-index) as well as the additional metrics above. Although the classification models were not trained to predict future disease, we explored their performance for future disease prediction, as previously described.^10^We used the classification model output (a number between 0 and 1) and evaluated the ability of this variable to predict future outcomes, using the *C*-index. For model evaluation, patients with moderate or severe valve disease for the valve being tested were excluded. For example, for evaluation of future significant MR prediction, patients with moderate or severe MR at baseline were excluded. We used a blanking period of 60 days following the index ECG when assessing for future rVHD prediction. Risk quartiles were defined (low, intermediate-low, intermediate-high, and high) based on outputs in the SHZS internal tuning set.

The survival models were further assessed in the following approaches. First, since the information on aetiology of MR was available in the BIDMC cohort, we compared the performance of the survival model in predicting primary MR vs secondary MR in the external test set. Second, we evaluated the change in the outputs of the survival models over time in the process of development of significant rVHD. Subgroup analysis was performed in the external test set across subgroups of sex and ethnicity. Lastly, a sensitivity analysis was performed in the BIDMC cohort, using outpatients with baseline ECG with or without baseline echocardiogram. Patients with at least 6-month follow-up were included. Patients with events in the first 6 months were excluded to reduce the number of patients who in fact had undetected prevalent VHD at the time of the index ECG.

### Model comparison

The performance of the survival models was compared with demographic and routinely measured echocardiographic parameters that have been previously reported to be predictive of rVHD progression. These parameters include age, left atrial volume and diameter, and mitral valve annulus diameter for MR,^20,21^ aortic annulus, sinotubular junction, and left ventricular end-diastolic dimension for AR,^4,22^ and right ventricular diameter, tricuspid annulus diameter, and left atrial diameter for TR.^5,6,23^ Left ventricular ejection fraction (LVEF) is a key echocardiography parameter and was therefore also included in a sensitivity analysis. Mitral valve annulus diameter was not measured routinely in patients without significant MR and therefore was substituted with left ventricular end-diastolic dimension in MR prediction models. Echocardiography variables were indexed to body surface area where appropriate in a sensitivity analysis. Cox models were fit using baseline variables alone, AI-ECG prediction alone, and both baseline and AI-ECG variables. Complete case analysis was used for multivariate Cox models. Sensitivity analysis was performed using a Fine and Gray model accounting for the competing risk of death. Continuous net reclassification index (NRI) was used to evaluate the additive value of the AI-ECG predictions to the baseline models. NRI with discrete risk thresholds was not used as this can be sensitive to the choice of risk thresholds.^24^ Additionally, the performance of the classification and survival models was compared for diagnosis using AUROC (DeLong’s test) and for prediction using the *C*-index (partial likelihood ratio test). For prediction, the classification model’s output was repurposed as a predictor, while the survival model used 5-year risk estimates.

### Imaging association analysis

To explore the biological plausibility of our findings, we investigated the correlation between AI-ECG predictions and echocardiography measurements in the external test set. Univariate correlation was performed between AI-ECG predictions and TTE parameters as previously described^25^ Analyses were adjusted for sex, age, and age squared using linear regression.

### Explainability analysis

After median beats were extracted using the BRAVEHEART ECG analysis software,^26^ two approaches were used to understand the ECG morphologies associated with predicted rVHD progression. First, a variational autoencoder (VAE) was trained using median ECG beats (details in Supplementary data online, Supplementary Methods). Variational autoencoder latent features were input into a linear regression with predicted rVHD progression as the output, and the top three most important features as assessed by the *t*-value were visualized by latent traversal.^27^ Second, using the median beats, we calculated the average waveform from the 10 000 ECGs with the lowest and highest AI-ECG predictions. The mean and standard deviation of these waveforms were then plotted.

### Statistical analysis

Continuous variables were expressed as mean ± standard deviation, and categorical variables were presented as numbers and proportions. Continuous data were compared using Student’s *t*-test, and categorical data were compared using χ^2^ test. Nested Cox models were compared with the likelihood ratio test, while non-nested Cox models were compared with the partial likelihood ratio test. A *P*-value of < .05 was considered statistically significant. Statistical analyses were performed with R 4.2.0 statistical package (R Core Team, Vienna, Austria) and Python (version 3.9).

## Results

### Patient characteristics

A total of 128 554 patients were identified from the SHZS cohort as the internal test set and 34 214 patients from the BIDMC cohort as the external test data set. The flow diagram of the study is shown in *Figure 1*.

In the SHZS cohort, at baseline, 10 776 (5.6%), 5961 (3.1%), and 10 453 (5.5%) ECG–TTE pairs had significant MR, AR, or TR, respectively. A total of 51 362 ECG–TTE pairs (27 551 patients) had a follow-up TTE at least 60 days after the index ECG. In patients with no or mild MR, AR, or TR, 830 (3.4%), 386 (1.5%), and 999 (4.0%) patients developed clinically significant MR, AR, or TR, respectively, after a median follow-up of 1.79 ± 2.17 years. In the BIDMC cohort, at baseline, 2365 (6.9%), 508 (1.5%), and 1979 (5.8%) had significant MR, AR, and TR, respectively. A total of 51 702 ECG–TTE pairs (17 565 patients) had a follow-up TTE at least 60 days after the index ECG. In patients with no or mild MR, AR, or TR, 1776 (11.6%), 460 (2.7%), and 2156 (13.7%) patients developed significant MR, AR, or TR, respectively, after a median follow-up of 4.28 ± 6.18 years. Clinical and echocardiographic characteristics of the included patients are presented in *Table 1*. Event rates by number of years of follow-up are provided in Supplementary data online, *Table S4*, while median days between ECG and baseline echo, and diagnosis of rVHD are provided in Supplementary data online, *Table S5*.

**Table 1:** Baseline characteristics

	SHZS	BIDMC	*P*-value
No. of subjects	128 554	34 214	
Age, years	56.19 (15.35)	62.20 (16.18)	<.0001
Male sex	69 857 (54.3)	17 620 (51.5)	<.0001
LVEF (%)	65.02 (6.85)	61.08 (13.51)	<.0001
Significant MR	6720 (5.2)	2365 (6.9)	<.0001
Significant AR	3970 (3.1)	508 (1.5)	<.0001
Significant TR	6291 (4.9)	1979 (5.8)	<.0001
Hypertension	-	20 991 (61.4)	
Previous MI	-	4871 (14.2)	
Smoker	-	6137 (17.9)	
Diabetes mellitus	-	9777 (28.6)	
Hyperlipidaemia	-	19 424 (56.8)	
**Ethnicity**			
White	-	23 759 (69.4)	
Black	-	4323 (12.6)	
Hispanic	-	1669 (4.9)	
Asian	128 404 (99.99)	1634 (4.8)	
Other/unknown	150 (0.001)	2829 (8.3)	

For continuous variables, mean (standard deviation) is shown unless otherwise stated, while for categorical variables, the *n* (%) is shown.

### Classification artificial intelligence-enhanced electrocardiography model for diagnosis of regurgitant valvular heart disease

First, we trained AI-ECG classification models for the diagnosis of significant (defined as moderate or severe) rVHD. Predictions of AI-ECG diagnosed significant MR, AR, or TR with an AUROC of 0.881 [95% confidence interval (CI) 0.876–0.887), 0.816 (95% CI 0.809–0.823), and 0.891 (95% CI 0.886–0.896), respectively. In the BIDMC external test set, the AUCs were 0.754 (95% CI 0.745–0.763), 0.714 (95% CI 0.691–0.738), and 0.771 (95% CI 0.759–0.781), respectively (*Table 2*).

**Table 2:** Model comparison—regurgitant valvular heart disease survival neural network vs classification model

	AI-ECG Survival model	AI-ECG Classification model	*P*-value
For prediction of future rVHDs evaluated with *C*-index			
Internal test set (SHZS)			
MR	0.774 (0.753–0.792)	0.755 (0.737–0.774)	<.0001
AR	0.691 (0.657–0.720)	0.665 (0.638–0.697)	<.0001
TR	0.793 (0.777–0.808)	0.765 (0.748–0.781)	<.0001
External test set (BIDMC)			
MR	0.698 (0.684–0.709)	0.668 (0.655–0.682)	<.0001
AR	0.647 (0.616–0.676)	0.612 (0.583–0.644)	<.0001
TR	0.702 (0.687–0.715)	0.682 (0.670–694))	<.0001
For diagnosis of prevalent rVHDs evaluated with AUROC			
Internal test set (SHZS)			
MR	0.893 (0.889–0.897)	0.881 (0.876–0.887)	<.0001
AR	0.845 (0.838–851)	0.816 (0.809–0.823)	<.0001
TR	0.910 (0.905–0.914)	0.891 (0.886–0.896)	<.0001
External test set (BIDMC)			
MR	0.777 (0.767–0.785)	0.754 (0.745–0.763)	<.0001
AR	0.751 (0.731–0.774)	0.714 (0.691–0.738)	<.0001
TR	0.805 (0.795–0.816)	0.771 (0.759–0.781)	<.0001

### Survival artificial intelligence-enhanced electrocardiography model for diagnosis and prediction of future significant regurgitant valvular heart disease

Subsequently, we trained an AI-ECG model using a survival neural network to diagnose existing and predict the future development of significant rVHD. The survival neural network AI-ECG models were superior to the classification models for both diagnosis and prediction of future rVHD (*Table 2* and Supplementary data online, *Tables S6* and *S7* and *Figures S1–S4*).

**Figure 2:**
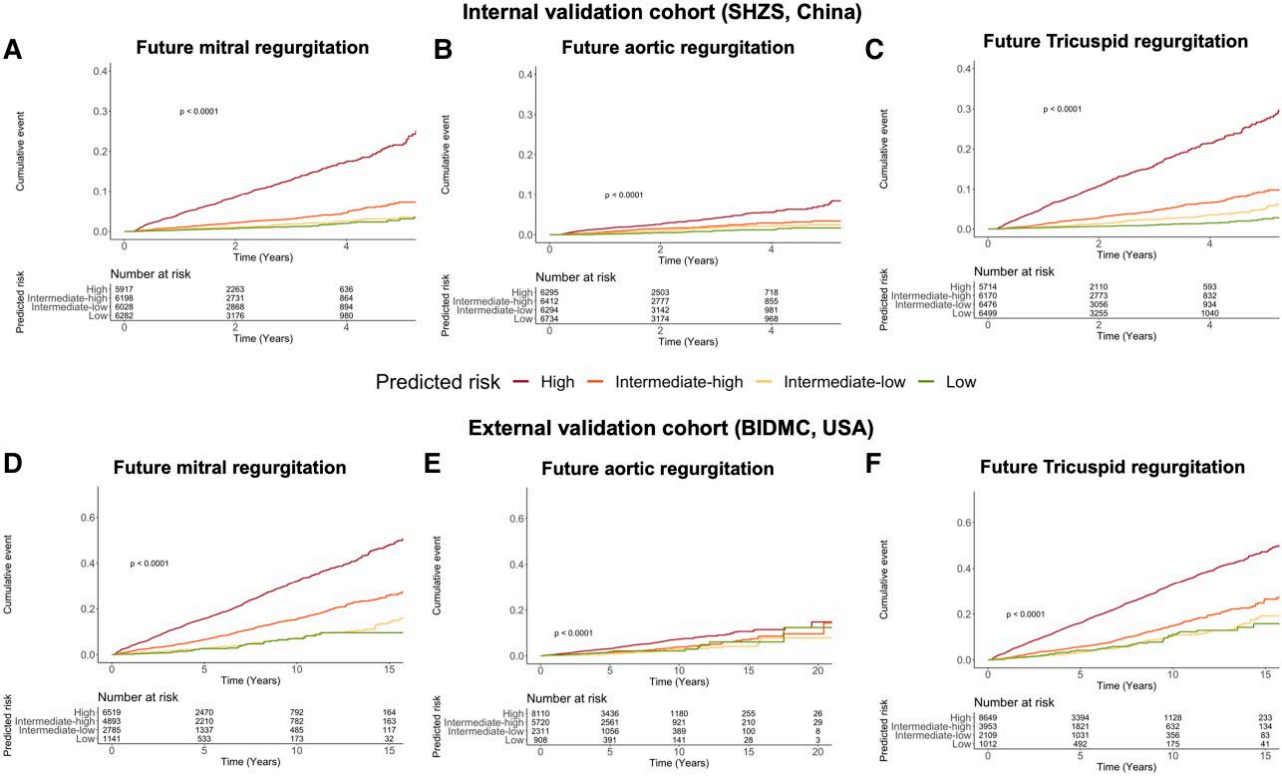
Progression regurgitant valvular heart diseases in different risk groups stratified by artificial intelligence-enhanced electrocardiography predictions in the internal test set (Shanghai Zhongshan Hospital, China) and external test set (Beth Israel Deaconess Medical Center, USA). Kaplan–Meier curves depicting the cumulative event rate (*Y*-axis) over time (*X*-axis) are shown. Shanghai Zhongshan Hospital: (*A*) mitral regurgitation; (*B*) aortic regurgitation; (*C*) tricuspid regurgitation. Beth Israel Deaconess Medical Center: (*D*) mitral regurgitation; (*E*) aortic regurgitation; (*F*) tricuspid regurgitation

In the internal test set, in individuals with no or mild rVHD at time of ECG, the AI-ECG survival models accurately predicted future significant (i.e. moderate or severe) MR (*C*-index 0.774, 95% CI 0.753–0.792), AR (0.691, 95% CI 0.657–0.720), and TR (0.793, 95% CI 0.777–0.808). In age- and sex-adjusted Cox models, the highest risk quartile had a hazard ratio (HR) of 7.6 (95% CI 5.8–9.9, *P* < .0001) for risk of future significant MR compared with the lowest risk quartile. For future AR and TR, the equivalent HRs were 3.8 (95% CI 2.7–5.5) and 9.9 (95% CI 7.5–13.0), respectively (*Figure 2A*–C). In the external test set, the ability of AI-ECG to predict future significant MR (C-index 0.698, 95% CI 0.684–0.709), AR (0.647, 95% CI 0.616–0.676), and TR (0.702, 95% CI 0.687–0.715) had a modest decrease in performance. Age- and sex-adjusted HRs comparing highest to lowest risk quartiles were 4.0 (95% CI 2.89–5.54), 1.45 (95% CI 0.88–2.37), and 3.0 (95% CI 2.3–3.9) for MR, AR, and TR, respectively (*Figure 2D–F*). Additional performance metrics are provided in Supplementary data online, *Tables S8* and *S9* and *Figures S3* and *S4*, and clinical characteristics by risk group are provided in Supplementary data online, *Table S10*. We additionally performed subgroup analyses in patients with/without LV systolic dysfunction, moderate/severe aortic stenosis, atrial fibrillation, and left bundle branch block (see Supplementary data online, *Tables S11–S13*) as well as a sensitivity analysis accounting for the competing risk of death (see Supplementary data online, *Table S14*).

**Figure 3:**
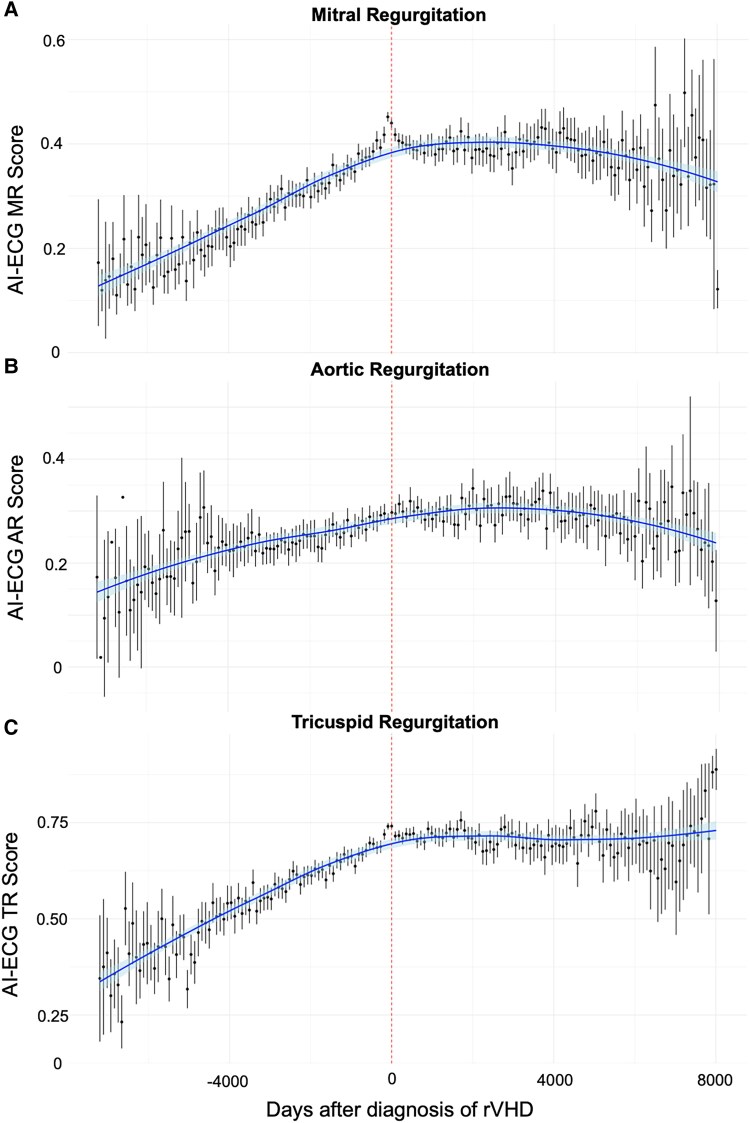
Artificial intelligence-enhanced electrocardiography scores increase over time before the development of significant regurgitant valvular heart diseases. Data are shown from the external test set (Beth Israel Deaconess Medical Center). Dotted vertical line indicates the first time of diagnosis of significant regurgitant valvular heart diseases on echocardiography. The predictions with 95% confidential intervals are present in bins of 90 days. (*A*) Mitral regurgitation; (*B*) aortic regurgitation; (*C*) tricuspid regurgitation

The survival model had superior performance for predicting significant secondary MR compared with primary MR (*C*-index 0.707, 95% CI 0.691–0.722 vs 0.659, 95% CI 0.608–0.707, *P* < .0001). Evaluation of AI-ECG predictions over serial ECGs showed that for patients with progression of rVHD during follow-up, the predictions of the survival models (AI-ECG score, predicted likelihood of developing significant rVHD) increased over time until clinically significant valve disease has developed, followed by a plateau (for TR) or a slight decrease (for MR and AR) (*Figure 3*).

**Figure 4:**
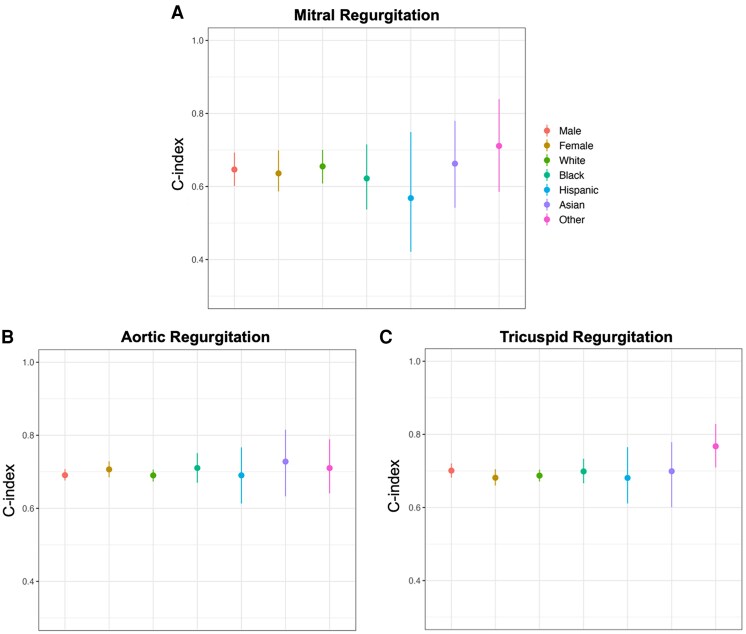
Artificial intelligence-enhanced electrocardiography performance in the Beth Israel Deaconess Medical Center external test set, stratified by sex and ethnicity. Artificial intelligence-enhanced electrocardiography performance is similar across sex and ethnic groups. The *C*-indices and 95% confidential intervals are presented. (*A*) Mitral regurgitation; (*B*) aortic regurgitation; (*C*) tricuspid regurgitation

The subgroup analysis revealed no significant differences in the performance of the survival models across various subgroups, including sex and ethnicity. Despite being developed in a Chinese population, the survival models exhibited comparable performance across all major ethnic groups within the external test set predominantly composed of white patients, supporting the generalizability of the models (*Figure 4* and Supplementary data online, *Table S15*). In order to mitigate the potential for selection bias as a driver for our findings, we performed a sensitivity analysis in the BIDMC cohort using outpatients with an ECG, with or without echocardiogram at baseline. We found robust performance in this sensitivity analysis (see Supplementary data online, *Table S16*).

### Artificial intelligence-enhanced electrocardiography is additive to echocardiographic predictors

When added to baseline Cox models (consisting of demographic and echocardiographic variables) for prediction of rVHD progression, the AI-ECG models were significantly additive in all cases. Most notably, AI-ECG markedly improved the prediction of severe TR 0.694 (95% CI 0.576–0.771) to 0.747 (95% CI 0.652–0.848) (*P* = .0005). Net reclassification indices ranged from 0.299 to 0.630, indicating the statistically significant and clinically relevant additive value of AI-ECG to demographic and echocardiographic factors (*Table 3* and Supplementary data online, *Tables S17* and *S18*).

**Table 3:** Comparison with clinical and echocardiographic parameters

Outcome	Baseline	AI-ECG	*P*-value^a^	Baseline + AI-ECG	*P*-value^b^	NRI
Moderate or Severe MR	0.729 (0.703–0.754)	0.707 (0.688–0.725)	<.0001	0.742 (0.711–0.771)	<.0001	0.353 (0.244–0.457)
Severe MR	0.708 (0.578–0.806)	0.711 (0.630–0.792)	.32	0.739 (0.625–.846)	.047	0.423 (−0.011–0.819)
Moderate or Severe AR	0.679 (0.637–0.719)	0.661 (0.636–0.685)	.04	0.705 (0.665–0.742)	< .0001	0.389 (0.308–0.570)
Severe AR	0.728 (0.691–0.770)	0.662 (0.627–0.697)	.001	0.741 (0.704–0.775)	< .0001	0.299 (0.134–0.450)
Moderate or Severe TR	0.714 (0.680–0.747)	0.702 (0.679–0.720)	.06	0.744 (0.715–0.773)	< .0001	0.490 (0.396–0.586)
Severe TR	0.694 (0.576–0.771)	0.744 (0.680–0.808)	.3415	0.747 (0.652–0.848)	.0005	0.630 (0.189–0.971)

NRI was evaluated when adding the AI-ECG prediction to the baseline model.

Baseline models:.

MR: age, sex, LA volume, LA dimension, LVEDD.

AR: age, sex, aortic sinus diameter, ascending aorta diameter, LVEDD.

TR: age, sex, RV diameter, LA volume.

AR, aortic regurgitation; LA, left atrial; LVEDD, left ventricular end-diastolic diameter; MR, mitral regurgitation; RV, right ventricular; TR, tricuspid regurgitation.

^a^Comparison between AI-ECG and baseline.

^b^Comparison between baseline + AI-ECG and baseline.

Cox models were fit using the variables listed and *C*-index was evaluated for the baseline model, artificial intelligence-enhanced electrocardiography prediction alone, and the combination of the baseline model and artificial intelligence-enhanced electrocardiography prediction.

### Biological exploration: artificial intelligence-enhanced electrocardiography identifies subclinical chamber remodelling to predict risk of regurgitant valvular heart disease

**Figure 5:**
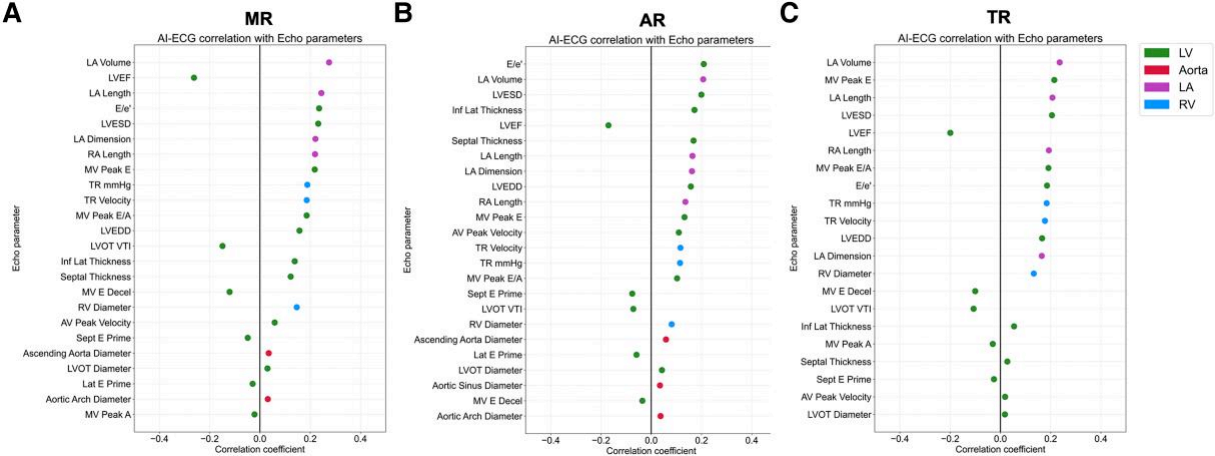
Correlations between artificial intelligence-enhanced electrocardiogram predictions with echocardiographic parameters. Associations demonstrate biologically plausible mechanisms for artificial intelligence-enhanced electrocardiography predictions. (*A*) Mitral regurgitation; (*B*) aortic regurgitation; (*C*) tricuspid regurgitation

To investigate structural associations underlying AI-ECG predictions, we correlated AI-ECG predictions with echocardiographic parameters in the BIDMC cohort. We found AI-ECG predictions were associated with subclinical chamber remodelling. For prediction of future significant MR, left atrial and left ventricular volumes and LVEF were strongly associated with AI-ECG predictions. For future AR, left ventricular volumes and wall thickness were most important, while for future TR, AI-ECG was associated with right and left atrial and ventricular sizes (*Figure 5*). Echocardiography parameter missingness is reported in Supplementary data online, *Table S19*.

### Explainable electrocardiography morphologies are associated with regurgitant valvular heart disease predictions

**Figure 6:**
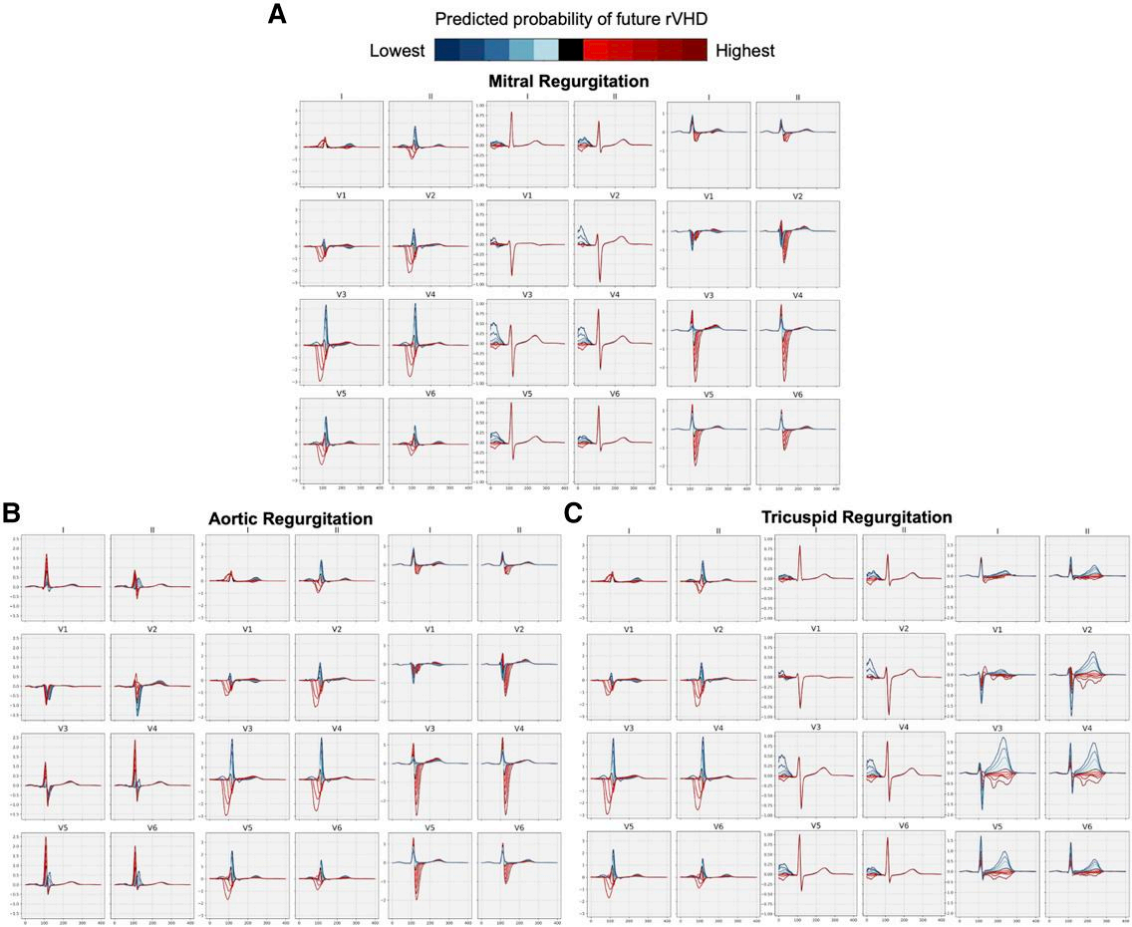
Variational autoencoder explainability analysis for artificial intelligence-enhanced electrocardiography regurgitant valvular heart disease predictions. Variational autoencoder electrocardiography morphologies associated with future regurgitant valvular heart disease are shown. The blue traces represent morphologies with lower probability of developing regurgitant valvular heart diseases progression, while the red traces represent morphologies with higher probability of developing regurgitant valvular heart diseases progression. *X* axes in ms; *Y* axes in mV. (*A*) Mitral regurgitation; (*B*) aortic regurgitation; (*C*) tricuspid regurgitation

**Figure 7:**
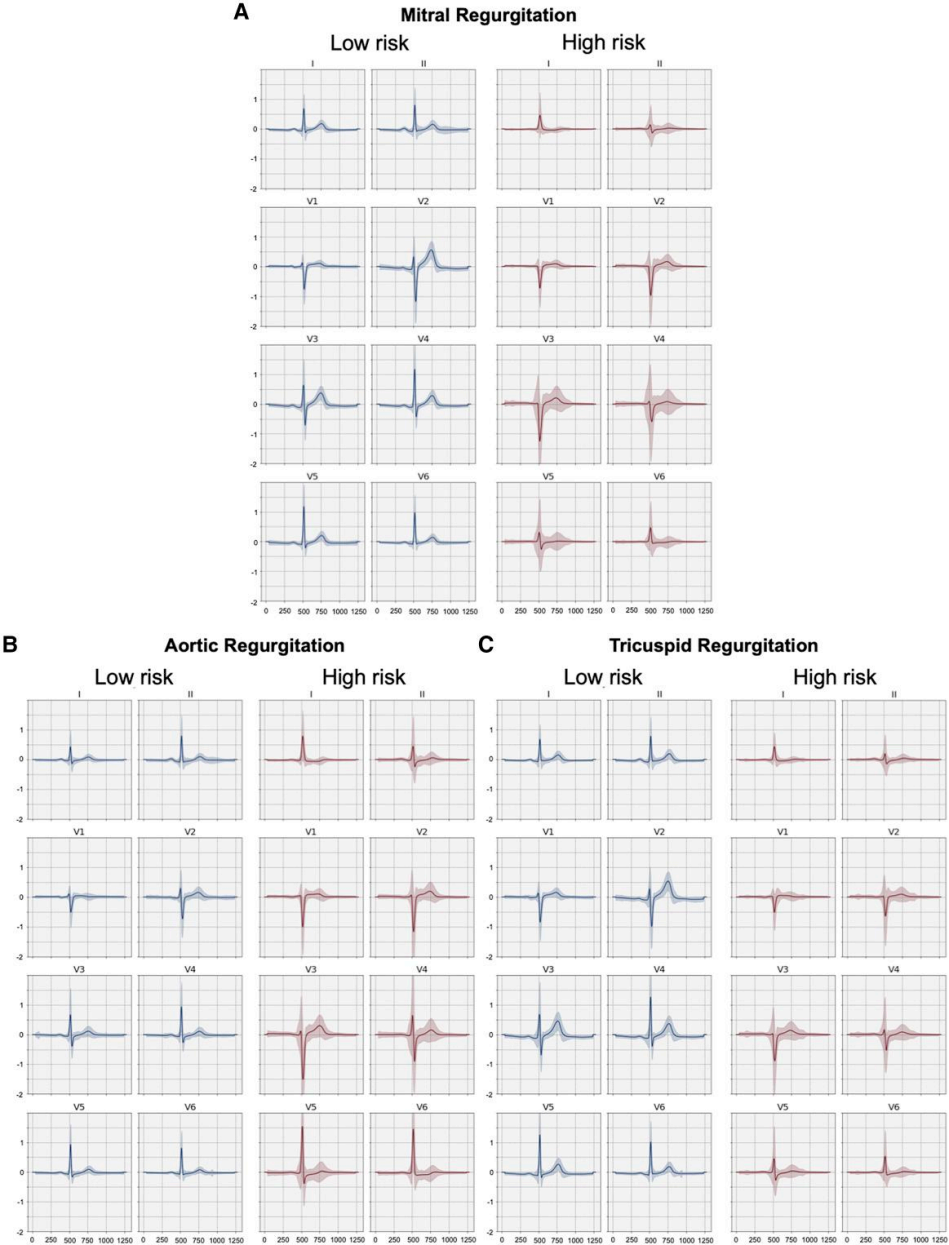
Median beat explainability. Average ± standard deviation (shaded region) electrocardiogram median waveforms for the 10 000 highest and lowest predicted regurgitant valvular heart disease risk electrocardiograms from the Beth Israel Deaconess Medical Center external test set. Red (high risk) waveforms show high predicted risk morphologies for each valve lesion, while blue (low risk) shows low predicted risk. *X* axes in ms; *Y* axes in mV. (*A*) Mitral regurgitation; (*B*) aortic regurgitation; (*C*) tricuspid regurgitation

We explored several methods to understand the biological underpinnings of our AI-ECG rVHD predictions. First, using a VAE, we visualized the features most correlated with the AI-ECG rVHD predictions in the BIDMC cohort (*Figure 6*). In the second approach, we used an average of median beats from the BIDMC test set (*Figure 7*).

Broader QRS, with left bundle branch block morphology, was an important feature for all three rVHD diagnoses. Interestingly, *P*-wave morphology was an important feature in regurgitant atrioventricular valves (MR and TR), but not AR. For left-sided regurgitant valve disease (AR and MR), QRS amplitude was an important feature. Finally, for TR, T-wave abnormalities were important features.

## Discussion

We developed the first AI-ECG models to predict future progression of rVHDs and tested the models in a transnational and ethnically distinct cohort. Predictions were biologically plausible and were mediated through detection of subclinical cardiac chamber remodelling. Our study demonstrated that AI-ECG could be utilized to guide surveillance echocardiography in patients at risk of future rVHD progression, to facilitate early detection and intervention (*Structured Graphical Abstract*).

### Artificial intelligence-enhanced electrocardiography accurately predicts regurgitant valvular heart disease progression

Our AI-ECG models accurately predicted the future development of significant MR, AR, and TR, despite a modest decrease in the *C*-indices in the external test set compared with the internal test set. This modest decrease in the external test set is consistent with prior work on other AI-ECG models for VHD,^11^ which could be attributed to the potential difference in patient characteristics, and clinical practice settings between study cohorts. In particular, we identified differences in disease incidence between the cohorts, which may be due to demographic differences. The BIDMC cohort represents a secondary care population, while SHZS patients are primarily from a primary care background and therefore are likely to have a lower incidence of disease. The high-risk group derived from our AI-ECG model demonstrated a significantly higher risk of developing rVHD progression compared with the low-risk group, indicating that our models can accurately risk stratify and identify patients who are most likely to experience progression of their regurgitant lesions, and may facilitate tailored management of these patients. Importantly, although our model was developed for the outcome of moderate or severe rVHDs, the performance was maintained for the outcome of severe rVHD alone (*Table 3*).

Previous studies have explored using echocardiographic risk factors to predict rVHD progression with logistic or Cox regression models,^4,5,21^ though no comprehensive multivariate predictive models have been established. Recent AI-ECG models have been developed to diagnose prevalent VHD, with the false-positive predictions being associated with having higher risk of progression compared with the true-negative predictions.^8,10^ Our study used a survival neural network architecture, which provides our model with the ability to predict time to rVHD progression without being constrained to a small number of time points, and allows us to use training data from patients that were censored and thus increasing the data available for model training. The strength of our approach is supported by the superior performance of our AI-ECG survival models compared with the Cox models incorporating the echocardiographic predictors.

Intriguingly, by training survival neural network models for both diagnosis and prediction of future incident rVHD, these models were superior for diagnostic applications when compared with the traditional AI-ECG classification models (*Table 2*). This difference is likely attributed to the training approach adopted by the AI-ECG survival neural network models, which integrate both follow-up echocardiographic data and initial diagnostic information, allowing more ECG–TTE pairs to be used in model training. For instance, individuals experiencing progression of rVHD are more likely to possess risk factors and early cardiac anatomical abnormalities, which could potentially be discerned by AI-ECG. In ‘learning’ the features important for future rVHD progression, the models appear to also be able to diagnose current rVHD more effectively than traditional classification models.

### Biological basis for artificial intelligence-enhanced electrocardiography prediction of regurgitant valvular heart disease progression

The underlying biological mechanism behind AI-ECG’s ability to predict rVHD progression likely involves identifying abnormal myocardial electrical activity occurring before or during the development of rVHD lesions. In our study, this subclinical chamber remodelling was assessed on the basis of echocardiographic parameters measured at the baseline examination and is depicted in *Figure 5*. Regurgitant VHDs can be categorized into primary and secondary aetiologies: primary rVHDs are characterized by intrinsic damage to the valve apparatus, whereas secondary rVHDs are caused by underlying conditions other than structural defects within the valve apparatus itself. As our study included a broad and unselected population, secondary rVHDs appear to be the more prevalent form.^28,29^

In secondary rVHDs, myocardial structural and functional remodelling typically precede rVHD development. These anomalies primarily include left ventricular dilatation and dysfunction with left atrial remodelling due to cardiomyopathies for MR and aortic diseases for AR, and RV and RA enlargement arising from left-sided heart disease and pulmonary hypertension for TR.^30^ Secondary MR in particular may be the consequence of left ventricular dilatation and dysfunction, and the aetiology could be both ischaemic and non-ischaemic. Our AI-ECG model may be detecting these early myocardial lesions and chamber remodelling, thereby distinguishing patients likely to experience rVHD progression from those unlikely.

In primary rVHDs, the chronic volume overload resulting from initial regurgitation prompts ventricular remodelling, which then alters the valve geometry, annular dimensions, and spatial relationships, further exacerbating the regurgitation.^31^ Chamber remodelling due to volume overload may occur early in the disease process and may be identified by AI-ECG models, offering predictive insights into future rVHD progression. However, chamber remodelling and associated electrical alterations may be subtle in the early stages of primary rVHDs, which may explain the reduced predictive performance compared with secondary rVHDs.

### Imaging association and explainability analyses support the biological plausibility of artificial intelligence-enhanced electrocardiography models

Overall, our imaging association analysis demonstrated correlations between AI-ECG predictions and both atrial and ventricular structural and functional traits across all types of rVHDs. Several of our findings are in line with previous reports regarding echocardiographic predictors of rVHD progression, such as the left atrial volume being the most strongly associated factor with MR progression^21^ and left atrial diameter demonstrating a correlation with TR progression.^32^

With the explainability analysis, for regurgitant atrioventricular valves (MR and TR), prolonged QRS duration, increased QRS, and decreased *P*-wave amplitude are the most significant indicators of future progression, indicating early ventricular and atrial anatomical and electrical remodelling, such as ventricular hypertrophy, reduced LVEF as well as intraventricular conduction delay and atrial fibrillation.^21^ In comparison, the most significant indicators for AR progression are exclusively related to the QRS complex, including both increased QRS amplitude and duration. Both left-sided rVHDs of MR and AR share common ECG features for high-risk AI-ECG predictions, including a deep and broad S-wave in V1 lead with monophasic broad lateral R-waves and negative T-waves, indicating left bundle branch block and reduced LVEF. Additionally, notable anterior T-wave inversion was an important feature in future TR predictions, suggesting the predictive value of right bundle branch block and right ventricular hypertrophy for progression.^5^

### Artificial intelligence-enhanced electrocardiography future regurgitant valvular heart disease prediction performance worse for aortic regurgitation than mitral regurgitation and tricuspid regurgitation

We observed that the performance of the AI-ECG models for future rVHD prediction is relatively diminished for AR in contrast to MR and TR. This disparity can be attributed primarily to distinct pathophysiological mechanisms underlying these rVHDs. For secondary causes, MR and TR typically involve dilatation of the corresponding mitral and tricuspid valve annuli associated with the left/right ventricles and left/right atria, whereas AR arises not only from left ventricular dilatation but also from distortion or dilation of the aortic root and/or ascending aorta.^33^ The aortic structures are devoid of significant electrical activity and thus less detectable by ECG. Furthermore, our association and explainability analyses suggest a relatively lesser involvement of the atrium in AR compared with MR and TR. The attenuated contribution of left atrial involvement in AR, coupled with the left ventricle’s greater resilience to pressure fluctuations, could potentially diminish the accuracy of prediction. Moreover, the progression trajectory of AR has been estimated to be milder compared with MR and TR,^4,5,20^ aligning with our findings from the same study population (*Figure 2*). This slower progression in AR implies a reduced frequency of positive instances during AI-ECG model training, which may also compromise predictive accuracy.

### Artificial intelligence-enhanced electrocardiography performs well across continents and ethnicity subgroups

Our study presents the first AI-ECG models for VHD that are trained from an East Asian population and tested in a geographically and ethnically distinct population. It is well known that ECG features vary by ethnicity.^34^ Specifically, there is evidence that prominent ethnic differences exist in ECG characteristics between White patients and Asians as well as a differential association with mortality.^35^ Moreover, variations exist in the prevalence of VHDs across different geographical regions and ethnic groups.^1,36^ However, our study demonstrated that, despite being trained using data from a homogeneous Asian population, the AI-ECG model was able to make predictions with high accuracy in an external testing American population, and furthermore without differential performance in different ethnic subgroups. These results are in line with a previous study demonstrating that in spite of racial differences in ECG data, a CNN trained on a homogeneous population generalized well to detecting low ejection fractions across several racial subgroups.^37^ Kaur *et al.*^38^ reported similar results showing that there were no significant differences observed between racial groups overall. Our study provides further evidence of the generalizability of AI-ECG for diagnostic and predictive purposes across different race groups. However, this generalizability is task-specific, and more evaluation of models in diverse subgroups of data sets is warranted.

### Clinical implications

Our study addresses a notable gap in the clinical management of rVHDs. It is anticipated that the recent advancements in AI-ECG technology will greatly facilitate the screening of rVHDs.^10,11,17^ During the screening process, most patients are likely to be found to have clinically insignificant rVHD. Presently, existing guidelines lack specific recommendations for managing these individuals.^7,39^ Our AI-ECG models stand poised to streamline the surveillance of these cases by providing enhanced diagnostic capabilities and facilitating personalized treatment plans. The AI-ECG models have potential for use either independently, like prior trials^40,41^ or in combination with other clinical and echocardiographic variables as described in our study.

Implementing AI-ECG models to predict the rVHDs progression and stratify patients into high- and low-risk categories would significantly impact clinical practice in several key aspects. Firstly, it would enable more intense follow-up for high-risk patients, ensuring timely monitoring of their condition and prompt intervention, if necessary, thus potentially reducing adverse outcomes such as heart failure or arrhythmias. Specifically, our models provide time-dependent predictions, thereby enabling dynamic adjustments to clinical evaluations and treatments in response to the evolution of disease. Secondly, the models could guide clinicians in selecting appropriate diagnostic methods tailored to each patient’s risk profile. For instance, in patients at risk of progression with multiple valve lesions, cardiac magnetic resonance assessment provides the advantage of individual measurements of severity independent of haemodynamic or filling status.^42^ Thirdly, through the identification of high-risk individuals, healthcare providers can deploy focused preventive interventions, such as lifestyle modifications and intensified medication regimens targeting primary diseases and comorbidities, aiming to decelerate or arrest the rVHD progression. As current guidelines and clinical practice in the treatment of VHDs reflect a shift towards earlier surgical or transcatheter intervention in appropriate candidates,^7,39^ our AI-ECG survival models could support personalized treatment planning by identifying high-risk patients who may benefit from earlier intervention. Lastly, AI-ECG may help improve resource utilization by reducing the frequency of echocardiographic surveillance in individuals with low-risk of progression.

### Limitations

Several limitations of our study need to be acknowledged. As both the ECG and TTE data were retrospectively collected from hospital-based cohorts, inherent patient selection bias may be introduced, potentially affecting the generalizability of our findings, especially since patients were likely selected for TTEs based on specific clinical indications in the BIDMC cohort. However, the robust performance in sensitivity analysis using patients with or without baseline echo is reassuring that selection bias did not affect the overall study findings. Future studies on non-symptom-driven ECGs in prospective cohorts will help provide a robust assessment of the model’s utility in a random screening population. The echocardiographic assessment of rVHDs relied on the interpretation provided by the cardiologist, introducing the potential for interobserver variability in the final diagnosis. Although the misdiagnosis rate is likely to be very low in the experienced centres participating in this study, it is possible that a small number of incident significant rVHDs were in fact initially misdiagnosed as mild or normal. Certain data such as patient comorbidities are not available in the internal test set; however, comprehensive assessments were conducted on the external test set. Unfortunately, we do not have data regarding the symptom status of the patients, nor the indication for the echocardiography investigation. The modest performance of our models, particularly in the external test set, suggests that the current models may not be ready for practical deployment. In particular, our AI-ECG models for AR prediction had reduced performance compared with MR and TR. This is may be due to distinct pathophysiological factors, such as the non-electrically active aortic structures. Further efforts are needed to refine and enhance the models’ performance before they can be effectively implemented in clinical practice. Due to limited patient numbers, we were unable to robustly assess the performance of the model in patients who had undergone prior valve surgery. While we have shown our AI-ECG models can predict the occurrence of future rVHD, echocardiography remains the gold standard for the diagnosis and characterization of VHD aetiology. Finally, while our models predicted rVHD progression, the clinical utility of these estimates for improving patient outcomes requires prospective evaluation in clinical trials.

## Conclusions

Artificial intelligence-enhanced electrocardiography can accurately diagnose prevalent and predict future development of significant MR, AR, and TR. For the first time, these findings were validated in an international and ethnically distinct cohort. Based on the observed performance characteristics of our model, this approach may serve as the basis for the development of a rVHD prediction programme, to facilitate early detection, timely intervention, and potentially preventative therapeutics, while also optimizing resource utilization by tailoring the frequency of echocardiographic surveillance based on risk of progression.

## Supplementary Material

ehaf448_Supplementary_Data

## Declarations

### Data Availability

The data in SHZS cohort are not publicly available due to ethical limitations but will be made available after a request for access to the corresponding author for the purpose of reviewing the study results. The underlying code for this study is not publicly available but may be made available to qualified researchers upon reasonable request to the corresponding author. Requests must include a clear research objective, a detailed analysis plan, and data requirements. If approved, the code will be provided under a collaboration agreement for non-commercial research purposes within 2 months.

### Pre-registered Clinical Trial Number

www.clinicaltrials.gov; unique identifier: NCT06475157.
