# Supplementary material for: Artificial intelligence-enhanced electrocardiography to predict regurgitant valvular heart diseases: an international study
Source: Eur Heart J. 2025 Jul 16;46(44):4823–37. doi: 10.1093/eurheartj/ehaf448 (PMC12634117; doi:10.1093/eurheartj/ehaf448)
Supplement: ehaf448_Supplementary_Data [file ehaf448_supplementary_data.docx]

**Artificial intelligence-enhanced electrocardiography to predict regurgitant valvular heart diseases: an international study**

**Supplementary Materials**

**Supplementary Methods**

**SHZS cohort further details**

The SHZS cohort includes a broader population, comprising many individuals who may not have clear cardiovascular symptoms or strong clinical indications. This open-access model to cardiovascular tests in Shanghai makes the SHZS cohort more representative of a community-based cohort than a strictly referral-based system, where patients are preselected based on suspected cardiac disease.

At the intra-hospital level (1) there are standardized protocols at Zhongshan Hospital that require both ECG and echocardiography to be performed in various routine and preoperative settings, regardless of whether patients have known or suspected cardiovascular disease. For instance, all patients scheduled for surgery - irrespective of the type of procedure or their cardiovascular history - must undergo both an ECG and an echocardiogram as part of their preoperative evaluation. This policy applies broadly, including patients undergoing non-cardiac surgeries. Since these assessments are conducted as standard hospital policy rather than being based on clinical suspicion of cardiovascular disease, our dataset naturally includes a substantial proportion of individuals without known or suspected cardiac conditions. (2) ECGs are also routinely performed in various non-symptom-driven scenarios, such as general health check-ups, pre-employment screenings, corporate wellness programs, and annual medical evaluations, through the department of Health Management Center. These practices further expand the population captured in our dataset, including individuals with no apparent cardiovascular concerns. (3) In the emergency department, ECGs are frequently performed as part of standard triage protocols, even for patients presenting with non-cardiac complaints. By incorporating patients across a broad range of clinical contexts, our dataset reflects a wide spectrum of cardiovascular risk profiles, including both high-risk individuals and a considerable number of asymptomatic or low-risk patients. This diverse sampling reduces the risk of referral bias and ensures our findings remain applicable to real-world clinical settings.

**Sample size**

Due to the very large datasets available for derivation and evaluation, formal sample size calculations were not performed.

**Model architecture and loss function**

The model was trained using the discrete-time survival approach described in (1). Specifically, the patients’ follow-up time is divided into a set of fixed intervals, and an estimated conditional hazard probability for each interval is calculated (probability of failure in that interval, given that the individual has survived at least to the beginning of the interval). For each time interval j, the loss function is defined as:

$$\sum_{i=1}^{d_{j}} ln\left( h_{j}^{i} \right)+\sum_{i=d_{j}+1}^{r_{j}} ln\left( {1-h}_{j}^{i} \right)$$

Where $h_{j}^{i}$ is the hazard probability for individual $i$ during time interval $j$, $r_{j}$ is the number of individuals that have not experienced failure or censoring before the beginning of the interval $j$, and $d_{j}$ is the number of individuals who have during the interval $j$. The overall loss function is the sum of the losses for each time interval.

The output of the neural network is an n-dimensional vector, with each element representing the predicted conditional probability of surviving that time interval $\left( {1-h}_{j}^{i} \right)$. An individual’s probability of surviving through the end of time interval $j$ is given by:

$$S_{j}= \prod_{i=1}^{j} (1-h_{i})$$

**Model training**

The output of the model is a predicted probability of survival within each discrete time-interval. The model was trained to account for events occurring 10 years from the time of the ECG. The model was selected based on the lowest tuning set loss and was evaluated on the unseen test set. Hyperparameter optimisation was performed using the SHZS tuning set. Models were trained for up to 50 epochs and the lowest tuning set loss of each training run used to evaluate model performance and select optimal hyperparameters. Models were trained using a single Nvidia RTX 6000 on Imperial College London’s high performance computing cluster. The Keras framework with a TensorFlow backend was used for neural network training and inference (2, 3).

**Variational auto-encoder training**

We trained a variational autoencoder (VAE) as previously described (4) using median ECG beats. The VAE consists of three components: the encoder, the decoder and the latent space. The encoder and the decoder are made up of one-dimensional convolutional layers with increasing filters and decreasing kernel sizes closer to the latent space. The latent space was restricted to 30 features at a maximum, although typically only a subset of these features was used by the model for the reconstruction. The model was trained to minimize both the median ECG reconstruction loss, defined by a symmetric mean absolute percentage error function, and the Kullback-Leibler divergence (KL loss). This second term is specifically added to the VAE model to ensure that the features generated by the model are generative and disentangled. An additional β-parameter was included as a weight on the KL-term to optimize the balance between the reconstruction loss and the latent factor interpretability. We tested beta values of 0.1, 0.25, 0.5, 1, 3, 5 and 10 and defined the best model at a β-parameter of 0.25 based on the Pearson correlation between the median and its reconstruction in the tuning dataset, as well as a visual inspection of the latent vector transversals.

We applied the VAE features to highlight the specific most important morphological features, as well as using a median beat explainability methodology. This dual approach provides not only average waveforms but also and understanding of which morphological components of the ECG were most important in deriving model predictions.

**Cox models proportional hazards**

Recent work suggests virtually all real-world clinical datasets will violate the proportional hazards assumptions if sufficiently powered and that statistical tests for the proportional hazards assumption may be unnecessary (5). In line with these recommendations, the proportional hazards assumption was not evaluated and the hazard ratio from our Cox models should be interpreted as a weighted average of the true hazard ratios over the follow-up period

**Echocardiography**

In both centres in this study, the assessment of all valves is included in the standard workup for echocardiography examinations, and the information could be extracted from the echo reports. In some very rare instances, assessment of a valve could not or was not performed. In these instances, a complete case analysis was performed with no imputation. The assessment of valvular heart disease severity was made by the clinician performing the scan and included any measurements taken as well as a subjective assessment. Unfortunately, we were unable to systematically examine each of the many thousand echocardiograms to assess which data was used to draw conclusions on the severity of VHD

**Table S1**: Major ECG abnormalities (SHZS)

| ECG abnormalities | Proportion (%) |
| --- | --- |
| AF | 3.10 |
| Supraventricular Tachycardia | 0.10 |
| First degree AVB | 2.57 |
| Second degree AVB | 0.14 |
| Third degree AVB | 0.11 |
| LBBB | 0.95 |
| RBBB | 4.29 |
| STEMI | 0.29 |
| LV hypertrophy | 3.35 |
| Hyperkalemia | 0.02 |
| Hypokalemia | 0.02 |
| Pacing | 0.80 |

**Table S2**: Major echocardiogram abnormalities for findings not included in study outcomes (SHZS)

| Echocardiogram abnormalities | Proportion (%) |
| --- | --- |
| LV systolic dysfunction (LVEF < 40%) | 2.19 |
| Hypertrophic cardiomyopathy | 0.28 |
| Ventricular septal defect | 0.05 |
| Atrial septal defect | 0.16 |
| Aortic stenosis (moderate or severe) | 1.09 |
| Mitral stenosis (moderate or severe) | 0.85 |

**Table S3**

Sensitivity analysis, comparison of AI-ECG survival model trained without exclusions compared to a model trained on a dataset excluding subjects with cardiac valve surgery, a prosthetic valve, or pacemaker/defibrillator implantation

|  | AI-ECG Survival model | AI-ECG Survival model trained with disease exclusions | P value |
| --- | --- | --- | --- |
| Internal test set (SHZS) |  |  |  |
| MR | 0.774 (0.753-0.792) | 0.749 (0.731-0.765) | P < 0.0001 |
| AR | 0.691 (0.657-0.720) | 0.649 (0.615-0.686) | P < 0.0001 |
| TR | 0.793 (0.777-0.808) | 0.781 (0.764-0.798) | P < 0.0001 |

**Table S4**

Table showing the percentage of each cohort with mild or no MR/AR/TR who go on to develop moderate or severe MR/AR/TR at 1, 2, 3 years in both cohorts and 5 years in the BIDMC cohort.

| **Disease** | **1 year** | **2 years** | **3 years** | **5 years** |
| --- | --- | --- | --- | --- |
| **SHZS** | | | | |
| MR | 1.77 | 4.47 | 9.12 | - |
| AR | 0.79 | 2.06 | 4.52 | - |
| TR | 1.98 | 5.26 | 10.84 | - |
| **BIDMC** | | | | |
| MR | 2.22 | 4.37 | 7.07 | 13.59 |
| AR | 0.4 | 0.93 | 1.67 | 3.34 |
| TR | 2.39 | 5.16 | 8.19 | 15.51 |

**Table S5**

Median days (IQR) between ECG and baseline echocardiogram, and ECG and diagnosis of rVHD in each cohort

|  | **ECG and echo baseline** | **ECG and diagnosis of rVHD** |
| --- | --- | --- |
| **SHZS** | | |
| MR | 1 (0-6) | 532 (239.25-1004) |
| AR | 1 (0-6) | 529 (238.25-928.75) |
| TR | 1 (0-6) | 545 (269-969) |
| **BIDMC** | | |
| MR | 15 (4-31) | 1507.5 (728.75-2860) |
| AR | 15 (4-31) | 1473.5 (626.25-2750) |
| TR | 15 (4-31) | 1461 (591.5-2733.25) |

**Table S6**

**Model performance for diagnosis of prevalent rVHDs using binary predicted AI-ECG survival model outcomes**

|  | Precision (PPV) | Recall (Sensitivity) | Specificity | Negative Predictive Value (NPV) | F1 Score |
| --- | --- | --- | --- | --- | --- |
| Internal test set (SHZS) | | | | | |
| MR | 0.216 (0.211-0.221) | 0.814 (0.805-0.823) | 0.837 (0.835-0.839) | 0.988 (0.987-0.989) | 0.341 (0.335-0.348) |
| AR | 0.107 (0.104-0.111) | 0.746 (0.733-0.760) | 0.802 (0.800-0.804) | 0.990 (0.989-0.991) | 0.187 (0.181-0.192) |
| TR | 0.241 (0.236-0.247) | 0.828 (0.818-0.837) | 0.866 (0.864-0.868) | 0.990 (0.989-0.990) | 0.374 (0.365-0.381) |
| External test set (BIDMC) | | | | | |
| MR | 0.148 (0.142-0.155) | 0.748 (0.730-0.766) | 0.680 (0.675-0.685) | 0.973 (0.971-0.975) | 0.248 (0.240-0.261) |
| AR | 0.028 (0.025-0.030) | 0.787 (0.749-0.822) | 0.579 (0.574-0.584) | 0.994 (0.993-0.995) | 0.053 (0.047-0.059) |
| TR | 0.116 (0.110-0.121) | 0.837 (0.820-0.853) | 0.606 (0.601-0.611) | 0.984 (0.982-0.985) | 0.203 (0.197-0.211) |

**Table S7**

**Model performance for diagnosis of prevalent rVHDs using continuous predicted outcomes**

|  | AUPRC | AUROC | Brier Score |
| --- | --- | --- | --- |
| Internal test set (SHZS) | | | |
| MR | 0.386 (0.374-0.399) | 0.893 (0.889-0.897) | 0.041 (0.041-0.042) |
| AR | 0.259 (0.245-0.273) | 0.845 (0.838-0.851) | 0.030 (0.030-0.030) |
| TR | 0.384 (0.374-0.394) | 0.906 (0.902-0.911) | 0.103 (0.101-0.104) |
| External test set (BIDMC) | | | |
| MR | 0.203 (0.190-0.215) | 0.777 (0.766-0.787) | 0.071 (0.070-0.072) |
| AR | 0.057 (0.046-0.071) | 0.751 (0.729-0.773) | 0.033 (0.032-0.033) |
| TR | 0.241 (0.225-0.258) | 0.804 (0.795-0.813) | 0.105 (0.103-0.107) |

**Table S8**

**Model performance for prediction of future rVHDs at the 3 year timepoint, using binary predicted AI-ECG survival model outcomes**

|  | Precision (PPV) | Recall (Sensitivity) | Specificity | Negative Predictive Value (NPV) | F1 Score |
| --- | --- | --- | --- | --- | --- |
| Internal test set (SHZS) | | | | | |
| MR | 0.266 (0.247-0.286) | 0.676 (0.643-0.708) | 0.754 (0.744-0.765) | 0.946 (0.940-0.952) | 0.382 (0.362-0.404) |
| AR | 0.085 (0.075-0.096) | 0.663 (0.614-0.710) | 0.590 (0.578-0.602) | 0.968 (0.962-0.973) | 0.151 (0.134-0.172) |
| TR | 0.298 (0.279-0.317) | 0.701 (0.671-0.729) | 0.739 (0.728-0.750) | 0.940 (0.933-0.946) | 0.418 (0.393-0.439) |
| External test set (BIDMC) | | | | | |
| MR | 0.257 (0.245-0.269) | 0.742 (0.721-0.762) | 0.541 (0.531-0.552) | 0.907 (0.899-0.915) | 0.382 (0.367-0.396) |
| AR | 0.048 (0.044-0.053) | 0.878 (0.845-0.907) | 0.226 (0.217-0.234) | 0.976 (0.969-0.982) | 0.092 (0.084-0.099) |
| TR | 0.264 (0.253-0.274) | 0.846 (0.830-0.861) | 0.388 (0.377-0.398) | 0.907 (0.897-0.916) | 0.402 (0.389-0.414) |

**Table S9**

**Model performance for prediction of future rVHDs at the 3 year timepoint, using continuous predicted outcomes**

|  | AUPRC | AUROC | Brier Score |
| --- | --- | --- | --- |
| Internal test set (SHZS) | | | |
| MR | 0.345 (0.317-0.382) | 0.771 (0.756-0.788) | 0.094 (0.091-0.098) |
| AR | 0.121 (0.102-0.147) | 0.680 (0.653-0.706) | 0.063 (0.061-0.065) |
| TR | 0.401 (0.371-0.436) | 0.788 (0.771-0.806) | 0.127 (0.121-0.132) |
| External test set (BIDMC) | | | |
| MR | 0.303 (0.285-0.323) | 0.690 (0.680-0.703) | 0.154 (0.150-0.158) |
| AR | 0.072 (0.061-0.084) | 0.623 (0.597-0.650) | 0.078 (0.076-0.079) |
| TR | 0.363 (0.346-0.383) | 0.697 (0.685-0.710) | 0.244 (0.238-0.249) |

**Table S10**

**Cohort clinical characteristics by risk groups in each cohort**

| **Mitral regurgitation risk groups** | | | | |
| --- | --- | --- | --- | --- |
| **SHZS** | | | | |
| **Risk category** | **High** | **Intermediate-high** | **Intermediate-low** | **Low** |
| **N subjects** | 5917 | 6198 | 6028 | 6282 |
| **Sex (M)** | 3557 (60.1) | 3327 (53.7) | 2764 (45.9) | 3611 (57.5) |
| **Age** | 61.87 (14.80) | 59.18 (14.43) | 54.84 (13.69) | 47.84 (12.53) |
| **LVEF** | 60.90 (10.03) | 65.39 (5.46) | 66.36 (4.68) | 66.83 (4.43) |
| **No AS** | 5477 (92.6) | 6053 (97.7) | 5971 (99.1) | 6247 (99.4) |
| **Mild AS** | 144 (2.4) | 61 (1.0) | 26 (0.4) | 14 (0.2) |
| **Mod/Sev AS** | 296 (5.0) | 84 (1.4) | 31 (0.5) | 21 (0.3) |
| **No/trace MR** | 4138 (69.9) | 5489 (88.6) | 5622 (93.3) | 6041 (96.2) |
| **Mild MR** | 1779 (30.1) | 709 (11.4) | 406 (6.7) | 241 (3.8) |
| **Mod/Sev MR** | 5917 (100.0) | 6198 (100.0) | 6028 (100.0) | 6282 (100.0) |
| **No/trace AR** | 4280 (72.3) | 5354 (86.4) | 5540 (91.9) | 6008 (95.6) |
| **Mild AR** | 925 (15.6) | 621 (10.0) | 376 (6.2) | 199 (3.2) |
| **Mod/Sev AR** | 712 (12.0) | 223 (3.6) | 112 (1.9) | 75 (1.2) |
| **No/trace TR** | 4439 (75.0) | 5543 (89.4) | 5574 (92.5) | 5884 (93.7) |
| **Mild TR** | 651 (11.0) | 435 (7.0) | 344 (5.7) | 336 (5.3) |
| **Mod/Sev TR** | 827 (14.0) | 220 (3.5) | 110 (1.8) | 62 (1.0) |
| **Incident mod/severe AS** | 95 (1.6) | 43 (0.7) | 24 (0.4) | 15 (0.2) |
| **Incident mod/severe MR** | 518 (8.8) | 160 (2.6) | 84 (1.4) | 68 (1.1) |
| **Incident mod/severe AR** | 356 (6.0) | 157 (2.5) | 99 (1.6) | 70 (1.1) |
| **Incident mod/severe TR** | 755 (12.8) | 269 (4.3) | 158 (2.6) | 83 (1.3) |
|  |  |  |  |  |
| **BIDMC** | | | | |
| **Risk category** | **High** | **Intermediate-high** | **Intermediate-low** | **Low** |
| **N subjects** | 6519 | 4893 | 2785 | 1141 |
| **Sex (M)** | 3908 (59.9) | 2461 (50.3) | 1282 (46.0) | 626 (54.9) |
| **Age** | 67.58 (13.68) | 62.95 (13.98) | 59.18 (13.98) | 54.01 (15.12) |
| **LVEF** | 58.02 (15.75) | 63.63 (12.12) | 64.06 (11.40) | 64.06 (11.33) |
| **No AS** | 5655 (86.7) | 4414 (90.2) | 2586 (92.9) | 1083 (94.9) |
| **Mild AS** | 307 (4.7) | 208 (4.3) | 102 (3.7) | 32 (2.8) |
| **Mod/Sev AS** | 557 (8.5) | 271 (5.5) | 97 (3.5) | 26 (2.3) |
| **No/trace MR** | 3370 (51.7) | 3437 (70.2) | 2127 (76.4) | 913 (80.0) |
| **Mild MR** | 3149 (48.3) | 1456 (29.8) | 658 (23.6) | 228 (20.0) |
| **Mod/Sev MR** | 6519 (100.0) | 4893 (100.0) | 2785 (100.0) | 1141 (100.0) |
| **No/trace AR** | 5155 (79.1) | 4122 (84.2) | 2419 (86.9) | 1010 (88.5) |
| **Mild AR** | 1220 (18.7) | 684 (14.0) | 327 (11.7) | 118 (10.3) |
| **Mod/Sev AR** | 144 (2.2) | 87 (1.8) | 39 (1.4) | 13 (1.1) |
| **No/trace TR** | 3608 (55.3) | 3358 (68.6) | 2033 (73.0) | 862 (75.5) |
| **Mild TR** | 2425 (37.2) | 1385 (28.3) | 685 (24.6) | 259 (22.7) |
| **Mod/Sev TR** | 486 (7.5) | 150 (3.1) | 67 (2.4) | 20 (1.8) |
| **Incident mod/severe AS** | 690 (10.6) | 379 (7.7) | 159 (5.7) | 52 (4.6) |
| **Incident mod/severe MR** | 1172 (18.0) | 439 (9.0) | 127 (4.6) | 38 (3.3) |
| **Incident mod/severe AR** | 256 (3.9) | 127 (2.6) | 56 (2.0) | 17 (1.5) |
| **Incident mod/severe TR** | 1367 (21.0) | 512 (10.5) | 177 (6.4) | 49 (4.3) |
| **Hypertension** | 4747 (72.8) | 3390 (69.3) | 1697 (60.9) | 587 (51.4) |
| **Previous MI** | 1203 (18.5) | 735 (15.0) | 352 (12.6) | 123 (10.8) |
| **Smoker** | 1436 (22.0) | 992 (20.3) | 454 (16.3) | 160 (14.0) |
| **Diabetes Mellitus** | 2444 (37.5) | 1681 (34.4) | 739 (26.5) | 246 (21.6) |
| **Hyperlipidemia** | 4207 (64.5) | 3086 (63.1) | 1641 (58.9) | 588 (51.5) |

| **Aortic regurgitation risk groups** | | | | |
| --- | --- | --- | --- | --- |
| **SHZS** | | | | |
| **Risk category** | **High** | **Intermediate-high** | **Intermediate-low** | **Low** |
| **N subjects** | 6295 | 6412 | 6294 | 6734 |
| **Sex (M)** | 3895 (61.9) | 3513 (54.8) | 3390 (53.9) | 3104 (46.1) |
|  |  |  |  |  |
| **Age** | 62.09 (14.41) | 60.53 (13.40) | 55.44 (12.89) | 47.11 (13.80) |
| **LVEF** | 59.30 (14.49) | 64.64 (7.03) | 66.01 (5.52) | 66.65 (4.98) |
| **No AS** | 5923 (94.1) | 6331 (98.7) | 6251 (99.3) | 6713 (99.7) |
| **Mild AS** | 139 (2.2) | 36 (0.6) | 26 (0.4) | 11 (0.2) |
| **Mod/Sev AS** | 233 (3.7) | 45 (0.7) | 17 (0.3) | 10 (0.1) |
| **No/trace MR** | 3507 (55.7) | 5171 (80.6) | 5695 (90.5) | 6277 (93.2) |
| **Mild MR** | 1317 (20.9) | 797 (12.4) | 448 (7.1) | 352 (5.2) |
| **Mod/Sev MR** | 1471 (23.4) | 444 (6.9) | 151 (2.4) | 105 (1.6) |
| **No/trace AR** | 5057 (80.3) | 5680 (88.6) | 5874 (93.3) | 6525 (96.9) |
| **Mild AR** | 1238 (19.7) | 732 (11.4) | 420 (6.7) | 209 (3.1) |
| **Mod/Sev AR** | 6295 (100.0) | 6412 (100.0) | 6294 (100.0) | 6734 (100.0) |
| **No/trace TR** | 4537 (72.1) | 5463 (85.2) | 5719 (90.9) | 6139 (91.2) |
| **Mild TR** | 659 (10.5) | 495 (7.7) | 396 (6.3) | 409 (6.1) |
| **Mod/Sev TR** | 1099 (17.5) | 454 (7.1) | 179 (2.8) | 186 (2.8) |
| **Incident mod/severe AS** | 76 (1.2) | 25 (0.4) | 22 (0.3) | 19 (0.3) |
| **Incident mod/severe MR** | 917 (14.6) | 348 (5.4) | 170 (2.7) | 130 (1.9) |
| **Incident mod/severe AR** | 178 (2.8) | 94 (1.5) | 73 (1.2) | 41 (0.6) |
| **Incident mod/severe TR** | 901 (14.3) | 395 (6.2) | 217 (3.4) | 170 (2.5) |
|  |  |  |  |  |
|  |  |  |  |  |
| **BIDMC** | | | | |
| **Risk category** | **High** | **Intermediate-high** | **Intermediate-low** | **Low** |
| **N subjects** | 8110 | 5720 | 2311 | 908 |
| **Sex (M)** | 4707 (58.0) | 2835 (49.6) | 1090 (47.2) | 441 (48.6) |
| **Age** | 67.52 (14.15) | 63.32 (13.92) | 58.49 (13.74) | 54.65 (15.81) |
| **LVEF** | 57.92 (16.46) | 61.61 (13.65) | 63.37 (12.14) | 63.73 (12.48) |
| **No AS** | 6991 (86.2) | 5229 (91.4) | 2210 (95.6) | 855 (94.2) |
| **Mild AS** | 357 (4.4) | 251 (4.4) | 65 (2.8) | 22 (2.4) |
| **Mod/Sev AS** | 762 (9.4) | 240 (4.2) | 36 (1.6) | 31 (3.4) |
| **No/trace MR** | 3804 (46.9) | 3613 (63.2) | 1701 (73.6) | 674 (74.2) |
| **Mild MR** | 3214 (39.6) | 1760 (30.8) | 542 (23.5) | 193 (21.3) |
| **Mod/Sev MR** | 1092 (13.5) | 347 (6.1) | 68 (2.9) | 41 (4.5) |
| **No/trace AR** | 6396 (78.9) | 4925 (86.1) | 2108 (91.2) | 820 (90.3) |
| **Mild AR** | 1714 (21.1) | 795 (13.9) | 203 (8.8) | 88 (9.7) |
| **Mod/Sev AR** | 8110 (100.0) | 5720 (100.0) | 2311 (100.0) | 908 (100.0) |
| **No/trace TR** | 4302 (53.0) | 3708 (64.8) | 1628 (70.4) | 652 (71.8) |
| **Mild TR** | 2996 (36.9) | 1728 (30.2) | 596 (25.8) | 222 (24.4) |
| **Mod/Sev TR** | 812 (10.0) | 284 (5.0) | 87 (3.8) | 34 (3.7) |
| **Incident mod/severe AS** | 834 (10.3) | 437 (7.6) | 90 (3.9) | 43 (4.7) |
| **Incident mod/severe MR** | 1522 (18.8) | 592 (10.3) | 145 (6.3) | 63 (6.9) |
| **Incident mod/severe AR** | 296 (3.6) | 115 (2.0) | 32 (1.4) | 17 (1.9) |
| **Incident mod/severe TR** | 1676 (20.7) | 687 (12.0) | 186 (8.0) | 85 (9.4) |
| **Hypertension** | 5900 (72.7) | 3901 (68.2) | 1304 (56.4) | 471 (51.9) |
| **Previous MI** | 1499 (18.5) | 952 (16.6) | 329 (14.2) | 96 (10.6) |
| **Smoker** | 1697 (20.9) | 1136 (19.9) | 417 (18.0) | 166 (18.3) |
| **Diabetes Mellitus** | 2897 (35.7) | 1924 (33.6) | 656 (28.4) | 243 (26.8) |
| **Hyperlipidemia** | 5285 (65.2) | 3615 (63.2) | 1298 (56.2) | 451 (49.7) |
|  | | | | |
| **Tricuspid regurgitation risk groups** | | | | |
| **SHZS** | | | | |
| **Risk category** | **High** | **Intermediate-high** | **Intermediate-low** | **Low** |
| **N subjects** | 5714 | 6170 | 6476 | 6499 |
| **Sex (M)** | 2915 (51.0) | 2731 (44.3) | 3241 (50.0) | 4935 (75.9) |
| **Age** | 61.78 (15.18) | 58.68 (14.32) | 54.32 (13.34) | 49.22 (12.95) |
| **LVEF** | 60.54 (11.24) | 64.66 (7.12) | 65.74 (5.68) | 66.17 (5.07) |
| **No AS** | 5437 (95.2) | 5977 (96.9) | 6341 (97.9) | 6401 (98.5) |
| **Mild AS** | 103 (1.8) | 64 (1.0) | 34 (0.5) | 39 (0.6) |
| **Mod/Sev AS** | 174 (3.0) | 129 (2.1) | 101 (1.6) | 59 (0.9) |
| **No/trace MR** | 3516 (61.5) | 5117 (82.9) | 5829 (90.0) | 6006 (92.4) |
| **Mild MR** | 1253 (21.9) | 758 (12.3) | 475 (7.3) | 308 (4.7) |
| **Mod/Sev MR** | 945 (16.5) | 295 (4.8) | 172 (2.7) | 185 (2.8) |
| **No/trace AR** | 4319 (75.6) | 5231 (84.8) | 5837 (90.1) | 6038 (92.9) |
| **Mild AR** | 874 (15.3) | 620 (10.0) | 401 (6.2) | 270 (4.2) |
| **Mod/Sev AR** | 521 (9.1) | 319 (5.2) | 238 (3.7) | 191 (2.9) |
| **No/trace TR** | 4838 (84.7) | 5682 (92.1) | 6059 (93.6) | 6166 (94.9) |
| **Mild TR** | 876 (15.3) | 488 (7.9) | 417 (6.4) | 333 (5.1) |
| **Mod/Sev TR** | 0 (0) | 0 (0) | 0 (0) | 0 (0) |
| **Incident mod/severe AS** | 72 (1.3) | 55 (0.9) | 43 (0.7) | 28 (0.4) |
| **Incident mod/severe MR** | 717 (12.5) | 267 (4.3) | 173 (2.7) | 134 (2.1) |
| **Incident mod/severe AR** | 299 (5.2) | 189 (3.1) | 123 (1.9) | 115 (1.8) |
| **Incident mod/severe TR** | 612 (10.7) | 208 (3.4) | 121 (1.9) | 58 (0.9) |
|  | | | | |
| **BIDMC** | | | | |
| **Risk category** | **High** | **Intermediate-high** | **Intermediate-low** | **Low** |
| **N subjects** | 8649 | 3953 | 2109 | 1012 |
| **Sex (M)** | 4428 (51.2) | 2108 (53.3) | 1331 (63.1) | 780 (77.1) |
| **Age** | 66.71 (13.70) | 61.24 (14.11) | 58.11 (14.40) | 56.33 (14.48) |
| **LVEF** | 59.07 (15.54) | 62.81 (12.66) | 63.18 (12.24) | 63.02 (12.39) |
| **No AS** | 7692 (88.9) | 3572 (90.4) | 1900 (90.1) | 936 (92.5) |
| **Mild AS** | 360 (4.2) | 158 (4.0) | 96 (4.6) | 26 (2.6) |
| **Mod/Sev AS** | 597 (6.9) | 223 (5.6) | 113 (5.4) | 50 (4.9) |
| **No/trace MR** | 4431 (51.2) | 2752 (69.6) | 1607 (76.2) | 741 (73.2) |
| **Mild MR** | 3423 (39.6) | 1048 (26.5) | 426 (20.2) | 243 (24.0) |
| **Mod/Sev MR** | 795 (9.2) | 153 (3.9) | 76 (3.6) | 28 (2.8) |
| **No/trace AR** | 6980 (80.7) | 3321 (84.0) | 1793 (85.0) | 856 (84.6) |
| **Mild AR** | 1498 (17.3) | 556 (14.1) | 274 (13.0) | 129 (12.7) |
| **Mod/Sev AR** | 171 (2.0) | 76 (1.9) | 42 (2.0) | 27 (2.7) |
| **No/trace TR** | 5020 (58.0) | 2898 (73.3) | 1664 (78.9) | 774 (76.5) |
| **Mild TR** | 3629 (42.0) | 1055 (26.7) | 445 (21.1) | 238 (23.5) |
| **Mod/Sev TR** | 0 (0) | 0 (0) | 0 (0) | 0 (0) |
| **Incident mod/severe AS** | 772 (8.9) | 295 (7.5) | 145 (6.9) | 78 (7.7) |
| **Incident mod/severe MR** | 1446 (16.7) | 324 (8.2) | 143 (6.8) | 64 (6.3) |
| **Incident mod/severe AR** | 313 (3.6) | 94 (2.4) | 50 (2.4) | 38 (3.8) |
| **Incident mod/severe TR** | 1643 (19.0) | 336 (8.5) | 121 (5.7) | 56 (5.5) |
| **Hypertension** | 6091 (70.4) | 2610 (66.0) | 1321 (62.6) | 616 (60.9) |
| **Previous MI** | 1508 (17.4) | 587 (14.8) | 291 (13.8) | 159 (15.7) |
| **Smoker** | 1801 (20.8) | 766 (19.4) | 371 (17.6) | 175 (17.3) |
| **Diabetes Mellitus** | 3013 (34.8) | 1285 (32.5) | 645 (30.6) | 265 (26.2) |
| **Hyperlipidemia** | 5515 (63.8) | 2429 (61.4) | 1263 (59.9) | 579 (57.2) |

**Table S11**

Subgroup analysis in subjects with impaired/preserved LV function or moderate/severe aortic stenosis

|  | LVEF > 50 | LVEF <50 | Mod/Sev AS |
| --- | --- | --- | --- |
| SHZS | | | |
| MR | 0.759 (0.740-0.779) | 0.798 (0.775-0.825) | 0.684 (0.538-0.838) |
| AR | 0.681 (0.651-0.722) | 0.699 (0.648-0.746) | 0.458 (0.276-0.652) |
| TR | 0.789 (0.769-809) | 0.795 (0.773-0.817) | 0.618 (0.505-0.742) |
| BIDMC | | | |
| MR | 0.695 (0.680-0.710) | 0.619 (0.591-0.642) | 0.666 (0.630-0.707) |
| AR | 0.604 (0.573-0.636) | 0.617 (0.558-0.684) | 0.589 (0.519-0.659) |
| TR | 0.677 (0.661-0.693) | 0.651 (0.626-0.675) | 0.694 (0.653-0.728) |

**Table S12**

**Number of ECGs with AF or LBBB in each cohort**

| Cohort | AF | LBBB |
| --- | --- | --- |
| SHZS | 2498 (9.1%) | 265 (1.0%) |
| BIDMC | 1814 (10.3%) | 725 (4.1%) |

**Table S13**

**Comparison of AI-ECG with ECG diagnosis of AF/LBBB for prediction of future valvular heart disease**

| Outcome | ECG – AF/LBBB | AI-ECG |
| --- | --- | --- |
| Moderate or Severe MR | 0.557 (0.528-0.584) | 0.707 (0.688-0.725) |
| Severe MR | 0.578 (0.456-0.697) | 0.711 (0.630-0.792) |
| Moderate or Severe AR | 0.524 (0.493-0.555) | 0.661 (0.636-0.685) |
| Severe AR | 0.572 (0.536-0.603) | 0.662 (0.627-0.697) |
| Moderate or Severe TR | 0.557 (0.531-0.588) | 0.702 (0.679-0.720) |
| Severe TR | 0.587 (0.484-0.698) | 0.744 (0.680-0.808) |

**Table S14**

Sensitivity analysis - using a Fine-Gray model to account for competing risk of death. C-index is reported (95% CI).

| Outcome | AI-ECG |
| --- | --- |
| Moderate or Severe MR | 0.699 (0.684 - 0.711) |
| Severe MR | 0.721 (0.676-0.766) |
| Moderate or Severe AR | 0.650 (0.621-0.679) |
| Severe AR | 0.664 (0.635-0.693) |
| Moderate or Severe TR | 0.699 (0.686 - 0.708) |
| Severe TR | 0.739 (0.706-0.772) |

**Table S****15**

BIDMC Subgroup analysis by Sex and ethnicity, concordance index (95% confidence interval)

| Subgroup | MR | AR | TR |
| --- | --- | --- | --- |
| Male | 0.691 (0.676-0.708) | 0.647 (0.602-0.693) | 0.701 (0.682-0.720) |
| Female | 0.707 (0.685-0.729) | 0.636 (0.587-0.699) | 0.681 (0.660-0.705) |
| White | 0.690 (0.673-0.706) | 0.655 (0.608-0.700) | 0.687 (0.671-0.703) |
| Black | 0.711 (0.670-0.751) | 0.622 (0.537-0.715) | 0.699 (0.666-0.733) |
| Hispanic | 0.690 (0.613-0.767) | 0.568 (0.422-0.749) | 0.681 (0.611-0.765) |
| Asian | 0.728 (0.633-0.815) | 0.663 (0.542-0.78) | 0.699 (0.601-0.779) |
| Other ethnicity | 0.710 (0.641-0.789) | 0.711 (0.586-0.839) | 0.767 (0.710-0.828) |

**Table S16**

Sensitivity analysis in the BIDMC cohort, including subjects with baseline ECG with or without baseline echocardiogram. Subjects with at least 6 months follow up are included. Subjects with events in the first 6 months were excluded to reduce the number of subjects who in fact had undetected prevalent valvular heart disease at the time of the index ECG. Sensitivity analysis N = 97870 subjects.

| Outcome | AI-ECG sensitivity analysis, C-index |
| --- | --- |
| Moderate or Severe MR | 0.759 (0.755-0.762) |
| Severe MR | 0.744 (0.733-0.756) |
| Moderate or Severe AR | 0.742 (0.723-0.760) |
| Severe AR | 0.753 (0.731-0.774) |
| Moderate or Severe TR | 0.757 (0.754-0.759) |
| Severe TR | 0.787 (0.777-0.797) |

**Table S17. Sensitivity analysis - Comparison with clinical and echocardiographic parameters**Cox models were fit using the variables listed and C-index was evaluated for the baseline model, AI-ECG prediction alone and the combination of the baseline model and AI-ECG prediction. NRI was evaluated when adding the AI-ECG prediction to the baseline model. In this sensitivity analysis, LVEF was added as a predictor and echocardiography parameters (other than LVEF) were indexed to BSA.

Baseline models:
MR: age, sex, LA volume, LA dimension, LVEDD, LVEF
AR: age, sex, aortic sinus diameter, ascending aorta diameter, LVEDD, LVEF

TR: age, sex, RV diameter, LA volume, LVEF

| Outcome | Baseline | AI-ECG | P value* | Baseline + AI-ECG | P value** | NRI |
| --- | --- | --- | --- | --- | --- | --- |
| Moderate or Severe MR | 0.737 (0.699-0.764) | 0.707 (0.688-0.725) | <0.0001 | 0.748 (0.72-0.777) | <0.0001 | 0.324 (0.220-0.448) |
| Severe MR | 0.741 (0.634-0.835) | 0.711 (0.630-0.792) | 0.076 | 0.736 (0.605-0.846) | 0.093 | 0.399 (-0.174-0.701) |
| Moderate or Severe AR | 0.695 (0.662-0.729) | 0.661 (0.636-0.685) | 0.001 | 0.712 (0.671-0.752) | <0.0001 | 0.362 (0.248-0.487) |
| Severe AR | 0.729 (0.69-0.769) | 0.662 (0.627-0.697) | 0.0001 | 0.748 (0.71-0.781) | <0.0001 | 0.268 (0.119-0.382) |
| Moderate or Severe TR | 0.73 (0.703-0.754) | 0.702 (0.679-0.720) | 0.002 | 0.751 (0.713-0.776) | <0.0001 | 0.492 (0.364-0.597) |
| Severe TR | 0.717 (0.617-0.828) | 0.744 (0.680-0.808) | 0.2197 | 0.757 (0.615-0.854) | 0.0003 | 0.574 (0.273-0.835) |

AR, aortic regurgitation; LA, left atrial; LVEDD, left ventricular end diastolic diameter; MR, mitral regurgitation; RV, right ventricular; TR, tricuspid regurgitation.

* indicates comparison between AI-ECG and baseline
** indicates comparison between baseline + AI-ECG and baseline

**Table S18 Cox model coefficients for baseline + AI-ECG models in Table S14**

| **Moderate MR** |  |  |  |  |  |  |  |
| --- | --- | --- | --- | --- | --- | --- | --- |
| **Variable** | **Coefficient** | **HR** | **HR (lower CI)** | **HR (upper CI)** | **Standard Error** | **Wald statistic** | **P value** |
| Age | 0.034 | 1.034 | 1.027 | 1.041 | 0.004 | 9.561 | <0.001 |
| Sex (female) | 0.206 | 1.229 | 1.043 | 1.448 | 0.084 | 2.469 | 0.014 |
| LA volume | 0.021 | 1.022 | 1.015 | 1.029 | 0.003 | 6.277 | <0.001 |
| LA dimension | 0.312 | 1.366 | 1.095 | 1.704 | 0.113 | 2.769 | 0.006 |
| LVEDD | 0.146 | 1.157 | 0.935 | 1.432 | 0.109 | 1.341 | 0.180 |
| LVEF | -0.012 | 0.988 | 0.982 | 0.995 | 0.003 | -3.510 | <0.001 |
| AI-ECG prediction | 1.216 | 3.373 | 2.406 | 4.729 | 0.172 | 7.051 | <0.001 |
| **Severe MR** |  |  |  |  |  |  |  |
| Age | -0.003 | 0.997 | 0.975 | 1.018 | 0.011 | -0.309 | 0.758 |
| Sex (female) | -0.337 | 0.714 | 0.390 | 1.308 | 0.309 | -1.090 | 0.276 |
| LA volume | 0.007 | 1.007 | 0.982 | 1.033 | 0.013 | 0.551 | 0.582 |
| LA dimension | 1.021 | 2.775 | 1.291 | 5.964 | 0.390 | 2.615 | 0.009 |
| LVEDD | 0.164 | 1.179 | 0.560 | 2.479 | 0.379 | 0.433 | 0.665 |
| LVEF | -0.016 | 0.984 | 0.961 | 1.007 | 0.012 | -1.387 | 0.165 |
| AI-ECG prediction | 1.042 | 2.835 | 0.857 | 9.381 | 0.611 | 1.707 | 0.088 |
| **Moderate AR** |  |  |  |  |  |  |  |
| Age | 0.023 | 1.023 | 1.014 | 1.032 | 0.004 | 5.204 | <0.001 |
| Sex (female) | -0.407 | 0.665 | 0.538 | 0.823 | 0.108 | -3.764 | <0.001 |
| Aortic sinus diameter | 0.172 | 1.188 | 0.753 | 1.873 | 0.232 | 0.741 | 0.458 |
| Ascending aorta diameter | 1.180 | 3.253 | 2.215 | 4.778 | 0.196 | 6.013 | <0.001 |
| LVEDD | 0.004 | 1.004 | 0.768 | 1.311 | 0.136 | 0.027 | 0.979 |
| LVEF | -0.002 | 0.998 | 0.991 | 1.005 | 0.004 | -0.519 | 0.604 |
| AI-ECG prediction | 2.640 | 14.008 | 6.795 | 28.874 | 0.369 | 7.152 | <0.001 |
| **Severe AR** |  |  |  |  |  |  |  |
| Age | 0.026 | 1.026 | 1.018 | 1.035 | 0.004 | 5.968 | <0.001 |
| Sex (female) | -0.286 | 0.752 | 0.602 | 0.938 | 0.113 | -2.531 | 0.011 |
| Aortic sinus diameter | -0.251 | 0.778 | 0.460 | 1.315 | 0.268 | -0.939 | 0.348 |
| Ascending aorta diameter | -0.652 | 0.521 | 0.315 | 0.863 | 0.257 | -2.533 | 0.011 |
| LVEDD | 1.404 | 4.073 | 3.088 | 5.372 | 0.141 | 9.942 | <0.001 |
| LVEF | -0.004 | 0.996 | 0.989 | 1.003 | 0.004 | -1.050 | 0.294 |
| AI-ECG prediction | 2.314 | 10.118 | 4.883 | 20.966 | 0.372 | 6.226 | <0.001 |
| **Moderate TR** |  |  |  |  |  |  |  |
| Age | 0.037 | 1.038 | 1.030 | 1.045 | 0.004 | 9.982 | <0.001 |
| Sex (female) | 0.133 | 1.142 | 0.972 | 1.342 | 0.082 | 1.612 | 0.107 |
| RV diameter | 0.527 | 1.694 | 1.373 | 2.090 | 0.107 | 4.913 | <0.001 |
| LA volume | 0.010 | 1.010 | 1.007 | 1.014 | 0.002 | 6.449 | <0.001 |
| LVEF | -0.012 | 0.988 | 0.982 | 0.994 | 0.003 | -4.122 | <0.001 |
| AI-ECG prediction | 1.546 | 4.695 | 3.376 | 6.528 | 0.168 | 9.192 | <0.001 |
| **Severe TR** |  |  |  |  |  |  |  |
| Age | 0.029 | 1.029 | 1.006 | 1.054 | 0.012 | 2.420 | 0.016 |
| Sex (female) | -0.105 | 0.901 | 0.531 | 1.528 | 0.270 | -0.388 | 0.698 |
| RV diameter | 1.088 | 2.967 | 1.587 | 5.549 | 0.319 | 3.406 | 0.001 |
| LA volume | 0.012 | 1.012 | 1.002 | 1.022 | 0.005 | 2.353 | 0.019 |
| LVEF | -0.001 | 0.999 | 0.981 | 1.018 | 0.010 | -0.069 | 0.945 |
| AI-ECG prediction | 1.938 | 6.942 | 2.311 | 20.853 | 0.561 | 3.453 | 0.001 |

**Table S19**

**Table reporting percentage of missing data for each variable in Figure 5**

| **Parameter** | **Percentage missing** |
| --- | --- |
| LVEDD | 12.3% |
| LVESD | 34.5% |
| Septal Thickness | 12.7% |
| Inf Lat Thickness | 12.8% |
| Lat E Prime | 39.0% |
| Sept E Prime | 40.0% |
| RV Diameter | 48.9% |
| LA Volume | 57.6% |
| LA Dimension | 14.2% |
| LA Length | 18.6% |
| RA Length | 19.2% |
| AV Peak Velocity | 16.7% |
| LVOT Diameter | 27.0% |
| LVOT VTI | 34.2% |
| MV Peak E | 15.4% |
| MV Peak A | 27.2% |
| MV Peak E/A | 27.2% |
| MV E Decel | 18.2% |
| TR Velocity | 24.2% |
| TR mmHg | 24.1% |
| Aortic Sinus Diameter | 15.0% |
| Ascending Aorta Diameter | 23.3% |
| Aortic Arch Diameter | 46.5% |
| LVEF | 4.3% |

**Supplementary Figure S1**ROC curves (blue) and precision-recall curves (red) for diagnosis of prevalent rVHDs using AI-ECG survival model (SHZS)

**Supplementary Figure S2**ROC curves (blue) and precision-recall curves (red) for diagnosis of prevalent rVHDs using AI-ECG survival model (BIDMC)

**Supplementary Figure S3
**ROC curves (blue) and precision-recall curves (red) for prediction of future rVHDs at the 3 year timepoint (SHZS)

**Supplementary Figure S4**ROC curves (blue) and precision-recall curves (red) for prediction of future rVHDs at the 3 year timepoint (BIDMC)

**References**

1. Gensheimer MF, Narasimhan B. A scalable discrete-time survival model for neural networks. PeerJ. 2019;7:e6257.

2. Chollet F. Keras 2015 [Available from: <https://keras.io>.

3. Martín A, Ashish A, Paul B, Eugene B, Zhifeng C, Craig C, et al. TensorFlow: Large-Scale Machine Learning on Heterogeneous Systems. 2015.

4. van de Leur RR, Bos MN, Taha K, Sammani A, Yeung MW, van Duijvenboden S, et al. Improving explainability of deep neural network-based electrocardiogram interpretation using variational auto-encoders European Heart Journal - Digital Health. 2022;3(3):390-404.

5. Stensrud MJ, Hernan MA. Why Test for Proportional Hazards? JAMA. 2020;323(14):1401-2.
